# Evidences for a New Role of miR-214 in Chondrogenesis

**DOI:** 10.1038/s41598-018-21735-w

**Published:** 2018-02-27

**Authors:** Vânia Palma Roberto, Paulo Gavaia, Maria João Nunes, Elsa Rodrigues, Maria Leonor Cancela, Daniel Martins Tiago

**Affiliations:** 1 Centre of Marine Sciences (CCMAR/CIMAR-LA), University of Algarve, 8005–139 Faro, Portugal; 2 PhD Program in Biomedical Sciences, DCBM, University of Algarve, 8005–139 Faro, Portugal; 3 Algarve Biomedical Center, University of Algarve, Campus de Gambelas, 8005–139 Faro, Portugal; 4 Department of Biomedical Sciences and Medicine, University of Algarve, 8005–139 Faro, Portugal; 5 Instituto de Investigação do Medicamento (iMed.ULisboa), Faculty of Pharmacy, Universidade de Lisboa, Av. Prof. Gama Pinto, 1649–003 Lisbon, Portugal; 6 Department of Biochemistry and Human Biology, Faculty of Pharmacy, Universidade de Lisboa, Av. Prof. Gama Pinto, 1649–003 Lisbon, Portugal

## Abstract

miR-214 is known to play a role in mammalian skeletal development through inhibition of osteogenesis and stimulation of osteoclastogenesis, but data regarding other vertebrates, as well as a possible role in chondrogenesis, remain unknown. Here, we show that miR-214 expression is detected in bone and cartilage of zebrafish skeleton, and is downregulated during murine ATDC5 chondrocyte differentiation. Additionally, we observed a conservation of the transcriptional regulation of miR-214 primary transcript *Dnm3os* in vertebrates, being regulated by Ets1 in ATDC5 chondrogenic cells. Moreover, overexpression of miR-214 *in vitro* and *in vivo* mitigated chondrocyte differentiation probably by targeting activating transcription factor 4 (Atf4). Indeed, miR-214 overexpression *in vivo* hampered cranial cartilage formation of zebrafish and coincided with downregulation of *atf4* and of the key chondrogenic players *sox9* and *col2a1*. We show that miR-214 overexpression exerts a negative role in chondrogenesis by impacting on chondrocyte differentiation possibly through conserved mechanisms.

## Introduction

Most of the elements of the vertebrate skeleton are built through endochondral bone formation from a cartilage anlage. This complex process, involving chondrocyte commitment, proliferation, differentiation and hypertrophy, is governed by tightly orchestrated genetic and epigenetic programs and its disruption leads to pathological consequences^1–3^. MicroRNAs (miRNAs) are small non-coding RNAs, usually transcribed by RNA Polymerase II (Pol II), which regulate gene expression by translational repression or by messenger RNA (mRNA) degradation^4,5^. miRNAs have emerged as important regulators of skeleton formation where they exert multiple levels of control from cell fate decision, to proliferation, differentiation and cellular activities^6–8^. In that sense, it is not surprising that skeletal key players, such as Runx2 and Sox9, are regulated by several miRNAs^7–9^. Particularly, miR-214 was shown to inhibit bone formation by regulating Atf4^10^, and to promote osteoclastogenesis by targeting Pten^11^. More recently, the potential use of miR-214 as a therapeutic target in skeletal disorders was evidenced when miR-214 was shown to transit from osteoclast-derived exosomes to osteoblasts, and inhibit bone formation through Atf4 blockage^12^. Interestingly, Atf4 not only is a master regulator of osteoblast function but also modulates chondrocyte proliferation and differentiation during endochondral bone formation^13,14^. Although the putative role of miR-214 in chondrogenesis remains generally unknown, it is now accepted that the *Dnm3* opposite strand (*Dnm3os*) transcript, which encodes miR-214 and miR-199a cluster^15^, is crucial for normal mouse skeletal development, including cartilage formation^16^. Moreover, miR-199a was reported to act as an inhibitor of chondrogenesis by targeting Smad1^17^. These reports suggest that a role for miR-214 in chondrogenesis is still to uncover.

In this study, we explored a putative role for miR-214 on cartilage formation. We found that miR-214 is expressed in the cartilage of zebrafish, and is downregulated during differentiation of murine ATDC5 chondrogenic cells. Also, we show that Ets1 activates *Dnm3os* promoter in undifferentiated cells. Upon miR-214 overexpression, *Atf4* expression concomitantly decreased, suggesting that this gene could also be a target of miR-214 in chondrogenesis. Supporting this notion, miR-214 gain-of-function in zebrafish was shown to impair cranial cartilage formation, and this was accompanied by a downregulation of *atf4* and of crucial cartilage markers, thus unveiling a negative impact of miR-214 on chondrogenesis.

## Results and Discussion

### Mir-214 expression is associated with skeleton formation of zebrafish

The cluster miR-199a-2/miR-214 is transcribed from the opposite strand of Dynamin 3 (*Dnm3*), in a common primary transcript called *Dnm3os*^15,16,18^. In mammalian models, this transcript was shown to be essential for normal growth and development of the skeleton^16^, and particularly, miR-214 was found to control both osteogenesis and osteoclastogenesis^10,11^. However, the putative role of miR-214 in chondrogenesis remains to be explored. In early development in zebrafish, miR-214 was previously shown to be expressed in somites and in the mesenchyme surrounding developing skeletal elements^15,19^. Although these studies pointed towards a possible role of miR-214 in skeletogenesis, they failed to demonstrate a clear association with tissue calcification.

#### miR-214 temporal expression correlates with skeletogenesis time-points

**Figure 1:**
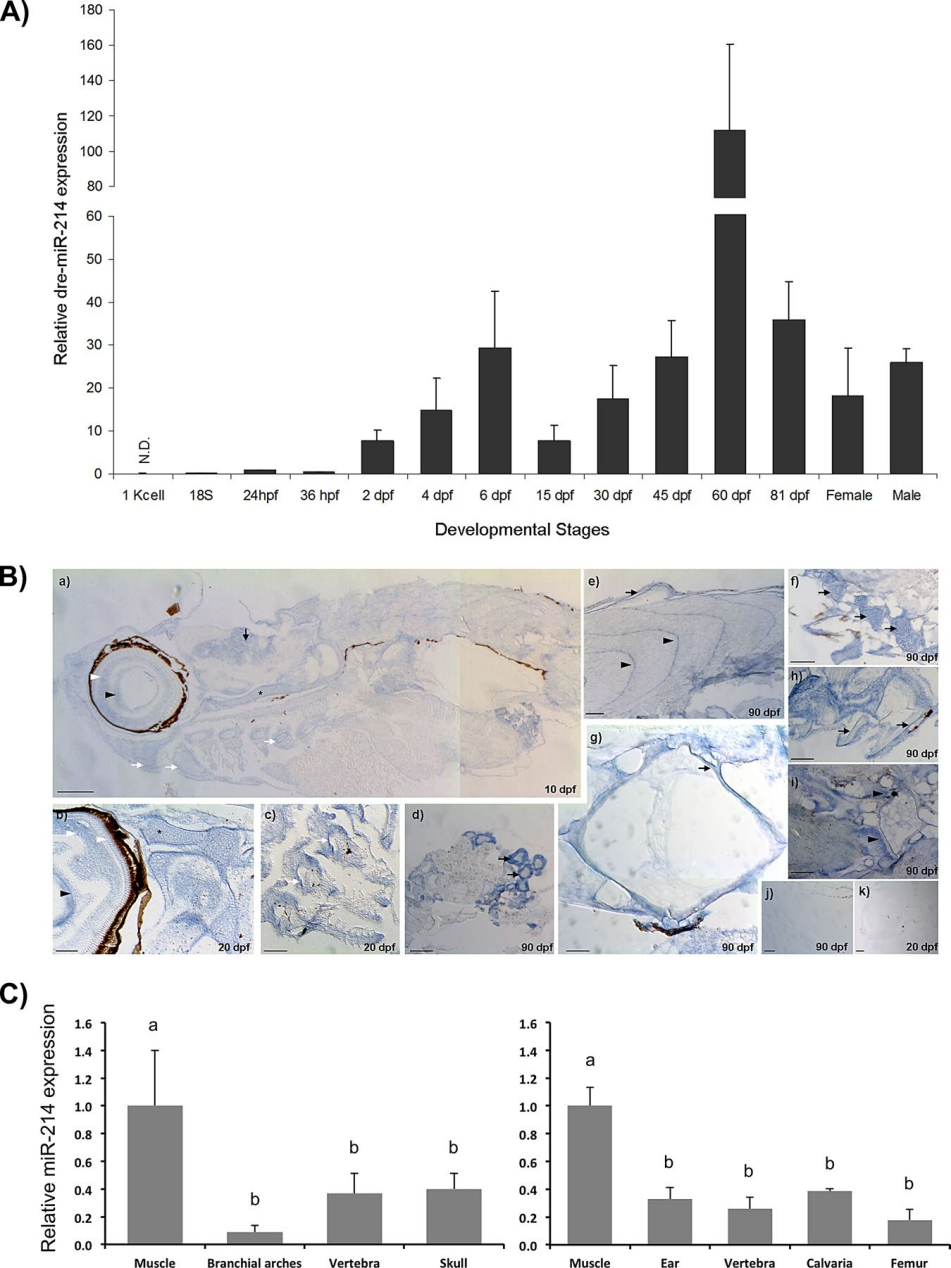
miR-214 expression correlates with skeletal elements in zebrafish. (**A**) Expression of miR-214 during zebrafish development, determined by miRNA qPCR. Values were normalized using zebrafish U6 small RNA and 24 hpf as reference sample and represent the mean ± s.d. of at least 3 independent replicates. *hpf* hours post fertilization, *dpf* days post fertilization, *N*.*D*. non-detected. Gap in the *y*-axis separates two different scales. (**B**) Detection of miR-214 by *in situ* hybridization in zebrafish with 10 (a), 20 (b, c, d) and 90 (e, f, g, h, i) dpf. From head to tail, miR-214 was detected in eye lens (arrowhead, a, b), retina (white arrowheads, a, b), brain (arrow, a), chondrocranium (asterisk, a, b), pharyngeal cartilage (white arrows, a, c), kidney (arrows, d), scales (arrow, e), muscle myotomes (arrowheads, e), cartilage in the base of pectoral fins (arrows, f), notochordal sheath (arrow, g), osteoid of haemal arches (arrows, h) and growth zones of vertebral body (arrowheads, i). Hybridization with negative control (scrambled) probe did not produce detectable signal, as observed in 90 and 20 dpf specimens (j and k, respectively). Scale bars: 0.2 mm for a, e, f and k; 0.1 mm for b, c, d, h, i and j; and 0.05 mm for g. (**C)** Relative expression of miR-214 in zebrafish (left panel) and mouse (right panel) adult tissues, determined by miRNA qPCR. Values were normalized using U6 small RNA and muscle as reference sample and represent the mean ± s.d. of at least 3 independent replicates (one-way Anova, different letters indicate statistical significance, p < 0.05). *B*. *arches*, Branchial arches. *dpf* days post fertilization.

To clarify this issue, we first analysed miR-214 expression throughout zebrafish development, from blastula to adulthood, focusing on crucial stages of skeletal formation (Fig. 1A). The following pattern of expression was observed: i) low levels of expression from 18-somite stage to 36 hpf; ii) progressive increase in expression from 2–6 days post fertilization (dpf), with a peak at 6 dpf (over 30-fold change comparing to 24 hours post fertilization, hpf), iii) decrease in miR-214 expression at 15 dpf; iv) progressive increase from 15 to 60 dpf (reaching 110-fold increase compared to 24 hpf); and v) a general decrease in miR-214 expression in young adults (at 81 dpf; similar levels observed in male and female) (Fig. 1A). As expected, miR-199a had a similar pattern of expression (Supplementary Fig. S1), consistent with the fact that both miRNAs are originated from the same transcript (*Dnm3os*). This is also in agreement with previous studies showing similar patterns of expression for miR-199 and miR-214 in different systems^15,20,21^. Interestingly, the patterns of expression found in this study were consistent with important time-points of skeleton formation during zebrafish development. Around 24 hpf, neural crest cells populate the two anterior pharyngeal arches, which will give rise to the craniofacial skeleton^22^. By 3 dpf, the onset of craniofacial calcification is concomitant with the appearance of major cartilaginous structures in the head, which continue to expand until 6 dpf^22,23^. From 6–15 dpf the craniofacial skeleton calcification substantially increases, and the calcification of the vertebrae is undergoing. At 30 dpf, all the skeletal structures are calcified^22,23^. The first mononucleated osteoclasts appear around 15 dpf, followed by multinucleated osteoclasts at 60 dpf, thus sustaining an active bone remodelling throughout adulthood^24^. The pattern of expression found for miR-214 during zebrafish development indicate that it might regulate distinct processes of skeleton formation, and further suggests that a tight regulation of miR-214 is required for a proper skeletal development. Nevertheless, one cannot disregard the putative involvement of miR-214 in other processes occurring simultaneously with skeletogenesis, not addressed in the scope of this study.

#### miR-214 spatial expression correlates with skeletal structures

To further understand a putative involvement of miR-214 in zebrafish skeletogenesis, the spatial component of miR-214 expression was analysed by *in situ* hybridization (Fig. 1B) at: i) 10 dpf, corresponding to the onset of vertebra calcification; ii) 20 dpf, when vertebra calcification is completed; and iii) 90 dpf, corresponding to young adult fish with active bone remodelling^23^. miR-214 was detected in both skeletal and non-skeletal components of zebrafish body throughout development, confirming previous studies discussed next^16,18^. Regarding non-skeletal components, miR-214 was detected in zebrafish brain, muscle and kidney (Fig. 1B), consistent with *Dnm3os* pattern of expression found during mouse development^18^. In zebrafish, we also detected miR-214 expression in eye lens and retina (Fig. 1B), consistent with previous data obtained in *X*. *laevies*, suggesting that miR-214 could also be associated with cell fate in zebrafish retina^25^. Overall, this miR-214 spatial distribution suggests a functional conservation in vertebrates. Concerning skeletal elements of zebrafish, miR-214 was found at sites where new bone is being formed, and also in several cartilaginous structures. Regarding cartilage, miR-214 expression was evident in the chondrocranium, in the pharyngeal cartilage and basal region of branchial filaments, in the ceratohyal, and in the basis of pectoral fins (Fig. 1B). As for newly forming bone, miR-214 was detected in arches and in the growth zones of the vertebral centra (Fig. 1B). MiR-214 was also expressed in the notochordal sheath and in scales (Fig. 1B). Our results are in agreement with previous data showing an association of *Dnm3os* with mouse cartilage^16^. However, to the best of our knowledge, miR-214 was never detected in cartilage. Thus, our results provide the first evidence indicating a possible role of miR-214 in chondrogenesis. In addition, miR-214 was detected in zebrafish vertebral column, reinforcing the idea that miR-214 is important for the onset of calcification during development^10,26^.

We further investigated the distribution of miR-214 in adult zebrafish calcified tissues, and argued if its expression could be comparable to other vertebrates. Thus, we analysed the expression of miR-214 in several calcified tissues from zebrafish (branchial arches, vertebra and skull) and mouse (cartilage from the ear, vertebra, calvaria and femur); given the known role of miR-214 in myogenesis, we decided to use muscle as a positive control^19,27^. We found high levels of miR-214 expression in all skeletal tissues, although the highest level was observed in the muscle of both zebrafish and mouse (Fig. 1C). This result suggested a functional conservation of miR-214 in skeletogenesis between zebrafish and mammals. The next set of experiments aimed at exploring a possible conservation of miR-214 transcriptional regulatory mechanisms.

### Dnm3os promoter contains regulatory elements associated to chondrogenesis

In the previous section we confirmed that miR-214 and miR-199a have similar temporal expression patterns, consistent with the fact that both miRNAs derive from the same transcript. Although *Dnm3os* is located on the opposite strand of a *Dnm3* intron, expression of both miRNAs and *Dnm3* gene was shown to be distinct in both zebrafish and mouse^15,18^, suggesting that *Dnm3os* regulatory transcription unit is independent from *Dnm3*. Since the transcriptional mechanisms regulating *Dnm3os* were poorly explored in previous studies, we decided to clarify this point.

#### Comparative sequence analysis of the Dnm3os putative promoter region

**Figure 2:**
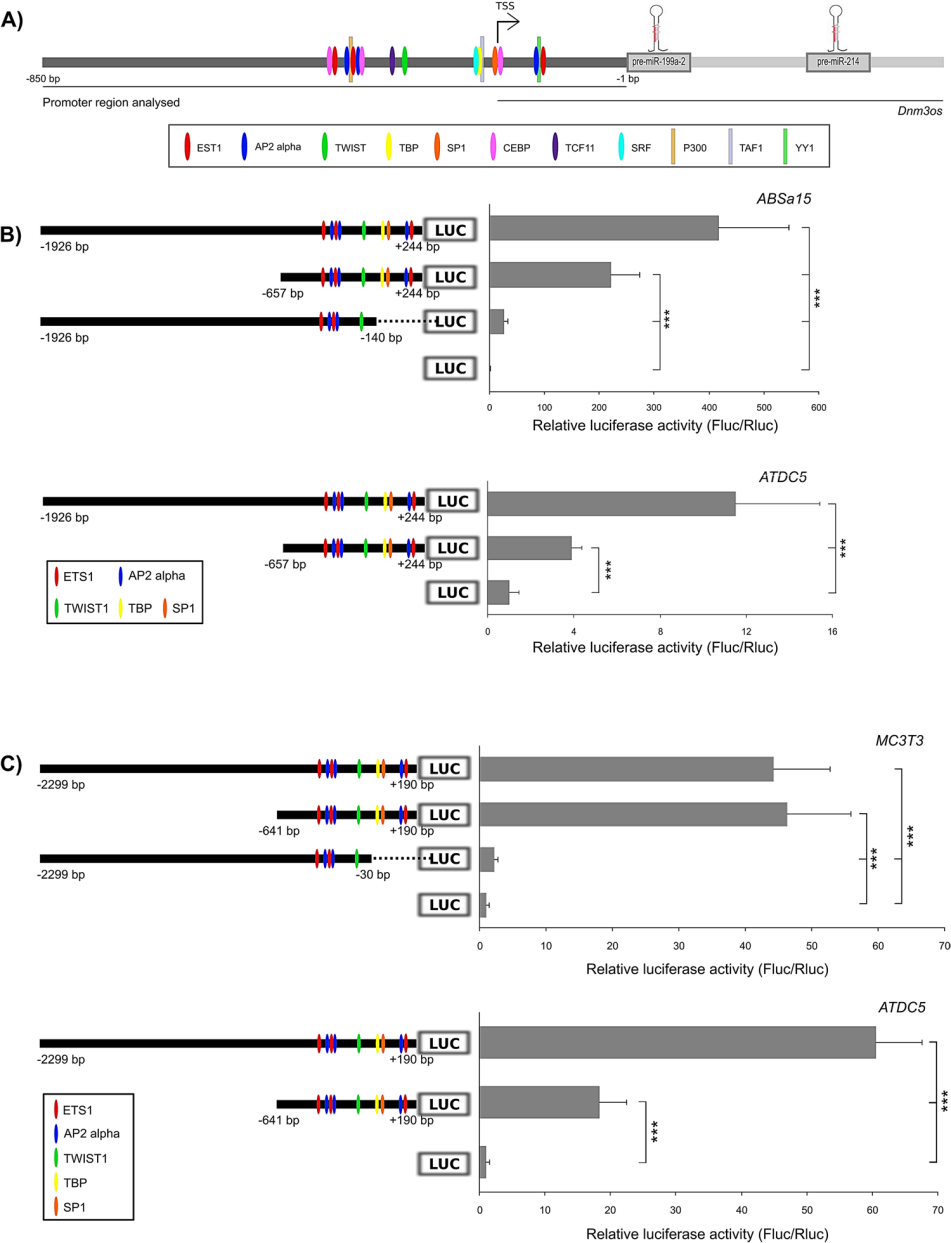
*Dnm3os* promoter is active in skeletal cell lines. (**A**) Schematic representation of *Dnm3os* gene (not scaled) and promoter region analysed (~850 bp). Putative promoter sequences of 8 vertebrates were aligned using CHAOS/DIALIGN, and then fed to ConTra v2: core match = 0.95, similarity matrix = 0.85, TRANSFAC database. TFBS predicted are indicated. Vertical lines indicate previously validated TFBS (by ChIP-assay) according to UCSC Genome browser. *TSS*, Transcriptional Start Site, based on human mRNA sequence; *bp*, base pairs. (**B**) Functional analysis of zebrafish *dnm3os* putative promoter activity in ABSa15 and ATDC5. Cells were transfected with zebrafish full (−1926 bp/+244 bp), partial (−657 bp/+244 bp), TATA-less (−1926bp/−140bp) promoter constructs, or promoter-less vector. (**C**) Functional analysis of human *Dnm3os* putative promoter activity in MC3T3 and ATDC5. Cells were transfected with human full (−2299 bp/+190 bp), partial (−641 bp/+190 bp), TATA-less (−2299bp/−30bp) promoter constructs, or promoter-less vector. For B) and C), a schematic representation of each construct and the respective putative TFBS are indicated on the left side. Values are the mean ± s.d. of at least 5 independent experiments; one-way Anova, ***p < 0.001.

We searched for conserved putative skeletal transcription factor binding sites (TFBS) in a 2.5 kilobase (kb) region upstream of pre-miR-199 (putative *Dnm3os* promoter) of human, mouse, Xenopus, medaka, stickleback, Tetraodon, fugu and zebrafish. Our analysis evidenced that the first ~850 base pairs (bp) of *Dnm3os* promoter (putative proximal promoter) were considerably more conserved, and contained most of the conserved TFBS, comparing to the more distant regions (putative distal promoter). Conserved binding sites for several TFs with known roles in skeletogenesis were identified: AP2alpha (transcription factor AP-2 alpha), CEBPs (CCAAT/enhancer binding proteins), ETS1 (ETS proto-oncogene 1, transcription factor), SP1 (family of transcription factors), SRF (serum response factor), TCF11 (also known as NFE2L1, nuclear factor, erythroid 2-like 1) and TWIST1 (twist family bHLH transcription factor 1) (Fig. 2A). Moreover, a conserved non-canonical TATA box (TATAT), present in seven out of eight species analysed, was identified 25 bp upstream the human transcriptional start site (TSS, GenBank accession NR_038397.2), and recognized as a putative binding site for TATA box binding protein, TBP (core = 0.90; matrix similarity = 0.75) (Fig. 2A and Supplementary Fig. S2A). Notably, the predicted binding sites for E1A binding protein p300 (P300), TAF1 RNA polymerase II TBP-associated factor (TAF1) and YY1 transcription factor (YY1), here predicted by Contra v2, were previously identified by ChIP-assays as *bona fide* binding sites (in Genome Browser at UCSC), thus supporting our in silico analysis. These results also revealed a remarkable conservation of the TFBS among vertebrates, further implying that *Dnm3os* transcription is regulated by mechanisms maintained throughout evolution.

#### Cloning and identification of a functional Dnm3os promoter

To test the functionality of human and zebrafish putative *Dnm3os* promoters, the corresponding genomic regions were cloned and inserted upstream the luciferase reporter gene in pGL3-Basic vector. TSS (+1) for human was deduced from the 5′end of the *Dnm3os* transcript variant 1 available at GenBank (NR_038397.2), whereas for zebrafish this information was not available. Therefore, we performed a 5′RACE PCR to determine the 5′end of zebrafish *Dnm3os*; the longest amplified sequence contained 244 bp upstream the first nucleotide of pre-miR-199a, and was considered as the TSS. Additionally, and considering our previous in silico analysis showing two distinct regions in putative *Dnm3os* promoter (proximal and distal), we have cloned genomic fragments corresponding to full (~2.5 kb; proximal plus distal) and partial promoters (~850 bp; proximal), plus TATA-less promoters, for human and zebrafish. Zebrafish promoter constructs in pGL3 were transfected into ABSa15, a fish bone-derived cell line (ECACC Ref. 13112201). In these cells the activity of the full promoter was 2 times higher comparing with the partial promoter (Fig. 2B), suggesting that the sequence from -1926 bp to -657 bp in zebrafish full promoter was sensitive to regulatory elements present in fish bone-derived cells. As expected, deletion of the putative TATA box significantly decreased luciferase activity to basal levels (Fig. 2B). The human promoter constructs were tested in mouse bone-derived cell line (MC3T3), where both full and partial promoters significantly increased luciferase expression to equivalent levels, and 45 times higher than empty vector (Fig. 2C). Again, the activity of the human promoter was almost abolished when the putative TATA box was deleted (Fig. 2C), indicating that the consensus TATA box here identified is functional in both species and crucial for *Dnm3os* transcription. Next we investigated promoter activities in an *in vitro* system that was extensively used in previous studies to investigate chondrocyte differentiation, i.e. the mouse teratocarcinoma-derived ATDC5 cell line^28^. Interestingly, full promoters of both zebrafish and human origins significantly increased luciferase activity to a higher extent than partial promoters (Fig. 2B,C), indicating that the distal region of these promoters should contain regulatory elements that are sensitive to transcriptional regulators present in ATDC5 cells. Our data indicates that both zebrafish and human promoters are active in chondrocyte- and osteoblast-like cell lines although to different extents, suggesting that *Dnm3os* is differentially regulated in distinct skeletal-derived cell lineages.

#### Transcriptional regulation of miR-214 in skeletal-related cell lines

**Figure 3:**
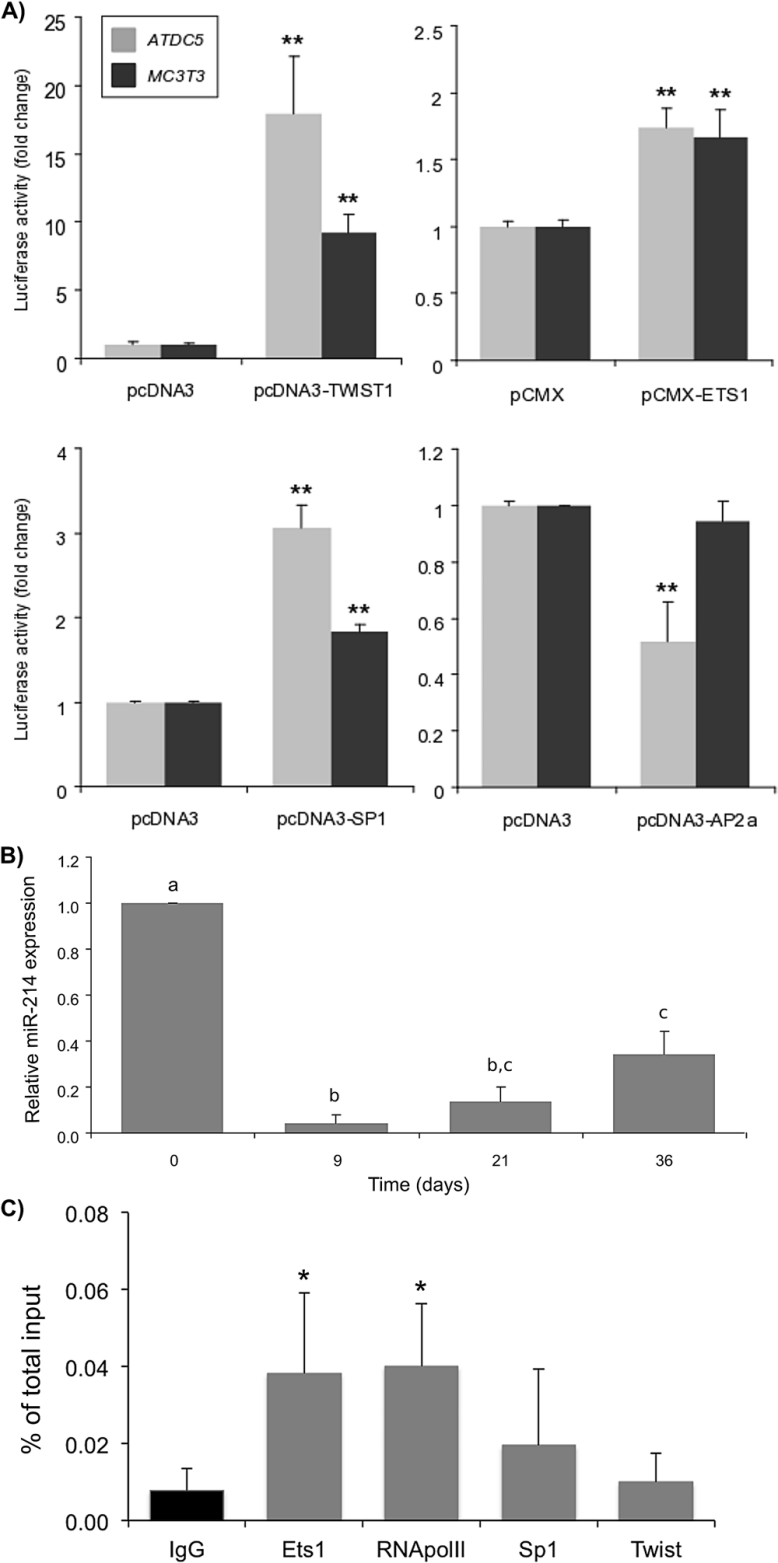
Analysis of miR-214 transcriptional activity in skeletal-derived cell lines. (**A**) Co-transfection of human *Dnm3os* promoter and skeletal TFs. ATDC5 and MC3T3 cells were co-transfected with full promoter construct and either pCMX-TWIST1, pCMX-ETS1, pCDNA3-SP1 or pCDNA3-AP2α, or empty vector (pCMX-PL2 or pCDNA3) as control. Results are indicated as fold change over the respective control empty vector (mean ± s.d. of at least 3 independent experiments; Student’s t-test, **p < 0.01). (**B**) Relative expression of miR-214 during ATDC5 cell chondrogenic differentiation. Levels of miR-214 were determined by miRNA qPCR, normalized using U6 small RNA expression and using day 0 (T0) as reference. Equal letters between two or more columns indicate values not statistically different, whereas different letters indicate values statistically different (mean ± s.d. of at least 3 independent replicates, one-way Anova, p < 0.05). (**C**) TF binding to *Dnm3os* proximal promoter analysed by Chromatin ImmunoPrecipitation (ChIP). Pre-cleared chromatin from undifferentiated ATDC5 cells was immunoprecipitated with either non-specific IgGs (IgG, negative control) or specific antibodies against Ets1, Sp1, Twist1, or RNA polymerase II (as positive control). After DNA recovery, the precipitates were assessed by qPCR. Data is expressed as percentage of total input and represent the mean ± s.d. of at least 3 independent experiments (one-way Anova, *p < 0.05).

To confirm our in silico analysis, expression vectors encoding TWIST1, ETS1, SP1 and AP2alpha were co-transfected with human full promoter constructs in ATDC5 and MC3T3 (Fig. 3A). While TWIST1, ETS1 and SP1 lead to an increase in *Dnm3os* promoter activity (from ~1.5-fold to ~10-fold; Fig. 3A), AP2alpha repressed *Dnm3os* promoter by 50% in ATDC5 cells; however, no effect was observed in MC3T3 (Fig. 3A), further supporting that *Dnm3os* transcriptional regulation is cell type-dependent. These data suggest that the conserved binding sites here identified for TWIST1, ETS1, SP1 and AP2alpha (Supplementary Fig. S2A and B) should contribute for *Dnm3os* transcription in skeletal-related processes. Importantly, co-transfection of zebrafish *Dnm3os* promoter with TWIST1 or ETS1 also led to an increase of luciferase activity (Supplementary Fig. S2C), thus supporting not only the functionality of the binding sites here identified, but also the conservation of *Dnm3os* transcriptional regulatory mechanisms across vertebrates.

#### miR-214 is downregulated during ATDC5 chondrogenic differentiation

To better understand miR-214 regulation in skeletal cells, in particularly in chondrogenesis, we used the ATDC5 chondrocytic cell line. First, we characterized the levels of miR-214 in critical stages of ATDC5 differentiation: i) at confluence, when cells are committed to chondrocyte lineage but are still chondroprogenitors (T0); ii) at the condensation stage / beginning of cartilaginous nodules formation (T9); iii) during nodule maturation, when chondrocytes are embedded in the matrix (T21); and iv) during mineralization, the later phase of differentiation (T36; Supplementary Fig. S3). miR-214 was highly expressed in confluent cells but strongly downregulated (over 10-fold change) during early (T9) differentiation (Fig. 3B). In subsequent stages (T21 and T36), miR-214 expression was somewhat increased, but its levels remained low comparing to T0 (6-fold lower in T36) (Fig. 3B). Not only this pattern of expression suggested that miR-214 could play a negative role on chondrogenesis, similar to what was previously shown during osteogenesis in MC3T3 cells^10^, but also that its levels should be tightly regulated during chondrocyte differentiation.

#### Ets1 stimulates Dnm3os transcription in undifferentiated ATDC5

Due to the pattern of expression of miR-214 found in ATDC5 cells, and in order to confirm our results on *Dnm3os* transcriptional regulation, we performed a ChIP analysis of *Dnm3os* promoter in undifferentiated ATDC5 cells using antibodies against ETS1, SP1, TWIST1 and RNA polymerase II (positive control). SP1 is not expressed in undifferentiated ATDC5 cells (data not shown), and thus was used as a negative control. Surprisingly, ETS1 but not TWIST1 was enriched in *Dnm3os* promoter (Fig. 3C), along with RNA polymerase II, thus confirming that *Dnm3os*, and miR-214, are expressed in undifferentiated ATDC5 cells. However, the levels expression found for ETS1 during ATDC5 differentiation (Supplementary Fig. S4) are not in line with miR-214 expression pattern (Fig. 3B). This discrepancy suggests that although ETS1 might contribute for miR-214 expression in undifferentiated ATDC5 cells, other TFs should also be determinant. Such TFs could be important for miR-214 down-regulation during ATDC5 differentiation. A good candidate for this downregulation could be AP2α, as evidenced in Fig. 3A. Nevertheless, one cannot exclude other TFs that were not yet investigated.

Still, we show for the first time that ETS1 is enriched in the *Dnm3os* promoter of ATDC5 cells in a region containing an evolutionary conserved ETS1 binding site (Figs 2, 3, Supplementary Fig. S2). Not only this result contributed to uncover the transcriptional regulation of *Dnm3os* (and miR-214) in ATDC5 cells, but also it highlighted a possible role for ETS1 in chondrogenesis, a topic that remains controversial. Ets1 is a pivotal TF in the speciation and migration of neural crest cells, which can originate cartilage. However, while *in vivo* data using Ets1-deficient mice evidenced the formation of ectopic cartilage nodules within the heart, suggesting that Ets1 can inhibit chondrocyte differentiation of cardiac neural crest cells^29^, *in vitro* studies in mouse mesencephalic neural crest cells showed that Ets1 is, on the contrary, able to promote chondrogenesis^30^. Moreover, contradictory results obtained in *X*. *laevis* model showed that both the overexpression and knockdown of Ets1 impaired cranial cartilage formation^31^. In fish, the role of Ets1 in skeleton formation remains to be explored. Nevertheless, in gilthead seabream this TF was shown to regulate the expression of two important regulators of cartilage formation: *bmp2* and cartilage-specific *s100*^32,33^. Altogether, these results indicate that ETS1 could be important to control vertebrate skeletogenesis through a putative regulation of *Dnm3os/*miR-214 expression.

### Negative effects of mir-214 on chondrogenesis

Although recent studies have demonstrated that miR-214 plays a role in skeleton formation^10,11^, the involvement of miR-214 in chondrogenesis remains unknown. However, data collected in our study suggests that miR-214 might have a role in chondrogenesis.

#### miR-214 mitigates the expression of chondrogenic markers during ATDC5 differentiation

**Figure 4:**
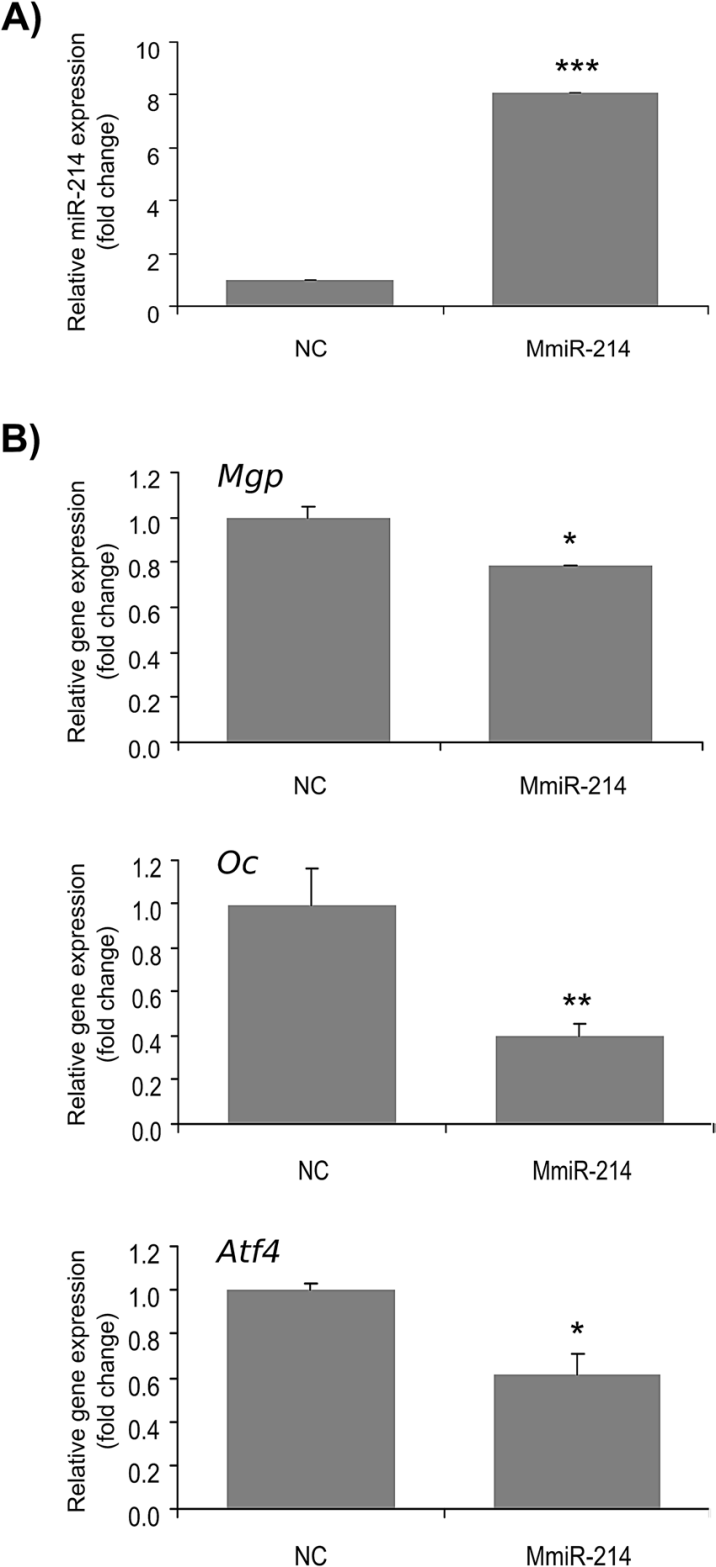
Effect of miR-214 on the expression of marker genes for chondrocyte differentiation in ATDC5 cells. ATDC5 cells were transfected with 50 nM of MmiR-214 or NC, and differentiation was induced at confluence (T0) for 14 days. (**A**) Levels of expression of miR-214 were determined by miRNA qPCR analysis and normalized using U6 small RNA. (**B**) Levels of expression of *Mgp*, *Oc* and *Atf4* were determined by standard qPCR and normalized using *Hprt1* housekeeping gene (similar results were obtained using *Hprt6* and *Gapdh* housekeeping genes; data not shown). Results are presented as fold change over NC. Asterisks indicate values statistically different from NC (data are the mean ± s.d. of at least 3 independent replicates; Student’s t-test, ***p < 0.001; **p < 0.01, *p < 0.05).

Gene expression analysis of ATDC5 cells indicated that miR-214 is differentially expressed during chondrocytic differentiation. The lower levels of expression during intermediate and later stages of differentiation suggest that miR-214 might be important to maintain chondrocytes in an undifferentiated condition. To further explore this possibility, and to get further insight into the role of miR-214 in chondrogenesis, we altered the expression of miR-214 in ATDC5 cells through gain and loss of function experiments. A miRNA mimic (MmiR-214) and an antagomiR (AmiR-214) with the respective controls (NC) were used to overexpress or down-regulate miR-214. ATDC5 differentiation occurred as previously reported^34,35^, as demonstrated by the expression of chondrogenic markers and von Kossa staining of cells (Supplementary Figs S3 and S4). QPCR analysis confirmed the altered expression of miR-214 in both experiments compared to NC at T14 (Fig. 4A, Supplementary Fig. S5). Surprisingly, at this stage the expression levels of *Col2a1*, *Col10a1*, *Tnap*, *Sox9* and *Sp7* were not affected by miR-214 overexpression (data not shown). On the contrary, forced expression of miR-214 significantly reduced *Mgp*, *Oc* and *Atf4* levels by approximately 20%, 60% and 40% respectively (Fig. 4B), suggesting that normal cell differentiation was most likely compromised. Although the role of Mgp in chondrogenesis is not fully understood, this protein was previously associated with chondrocyte proliferation and apoptosis^35^, being recognized as one of the main markers of chondrogenic differentiation *in vivo* and *in vitro*^35,36^. Conversely, Oc is mainly synthesized in osteoblasts and associated with bone formation, though its expression was also detected in chondrocytes and vascular smooth muscle cells, especially during mineralization^37^. In fact, Oc overexpression in ATDC5 was previously shown to stimulate cell differentiation and mineralization^37^. Therefore, in our experimental conditions, down-regulation of both Oc and Mgp upon miR-214 overexpression should represent a drawback in the differentiation process, probably with consequences at mineralization. Nevertheless, *Mgp* and *Oc* were still down-regulated upon loss-of-function of miR-214 (Supplementary Fig. S5), indicating that miR-214 mode of action in chondrogenesis might be more complex. Accordingly, precisely in the later stage of differentiation, the expression of miR-214 was slightly increased in WT cells (Fig. 3B), when *Mgp* and *Oc* levels are higher. Altogether, these data suggest that a tight regulation of miR-214 levels is required for a proper control of molecules impacting on chondrogenesis, such as Mgp and Oc, and consequent normal chondrocyte differentiation. Since bioinformatic analysis did not indicate *Mgp* or *Oc* as direct targets of miR-214 (data not shown), down-regulation of these genes was most likely indirect. On the contrary, the repression of *Atf4* by miR-214 overexpression in ATDC5 cells is likely a direct effect, since this regulatory mechanism was previously demonstrated in osteoblasts^10^ and upon antagomiR transfection *Atf4* levels were restored to those found in the control (Supplementary Fig. S5). Atf4 is a crucial player in chondrogenesis and its ablation in mouse (*Atf4*^*−/−*^) altered both proliferative and hypertrophic growth plate zones through control of Indian hedgehog (Ihh) expression^14^. Moreover, *Atf4* overexpression in chondrocytes of mouse *Atf4*^*−/−*^; *Col2a1-Atf4* double mutants positively regulated osteoblast differentiation during development through a paracrine mechanism also involving Ihh^38^. Atf4 is a TF essential for the regulation of osteoblast differentiation and bone development^13^, known to drive the expression of Sp7, through a PTH-dependent mechanism^39^, and Oc, by cooperative interaction with Runx2 and Satb2^40,41^. Atf4 is also one of the regulators of the neural crest cells migration, in cooperation with Sox9^42^. Thus, based on the important role of Atf4 on chondrogenesis, and the inverse patterns of expression of *Atf4* and miR-214 during ATDC5 differentiation (Supplementary Fig. S4 and Fig. 3B), we propose that Atf4 could be a target of miR-214 in ATDC5 cells, probably contributing to a mitigation of cell differentiation. However, miR-214 is known to regulate genes and pathways with important functions in chondrogenesis and alternative targets should be considered. For instance, miR-214 regulates the Hedgehog pathway in zebrafish by targeting *sufu*^19,43^ and was shown to regulate the WNT pathway through direct regulation of β-Catenin in humans^44^. Importantly, both targets have conserved binding sites for miR-214 (data not shown). To further explore this possibility, we tested the effect of miR-214 overexpression on Hh pathway by assessing the levels of a universal marker for activation of this pathway, Patched 1 (*Ptch1*). Indeed, *Ptch1* levels were significantly increased in ATDC5 cells overexpressing miR-214 compared to control cells (Supplementary Fig. S6), suggesting that this pathway could also be involved in miR-214 effects in chondrogenesis.

#### miR-214 overexpression impairs zebrafish cartilage formation *in vivo*

**Figure 5:**
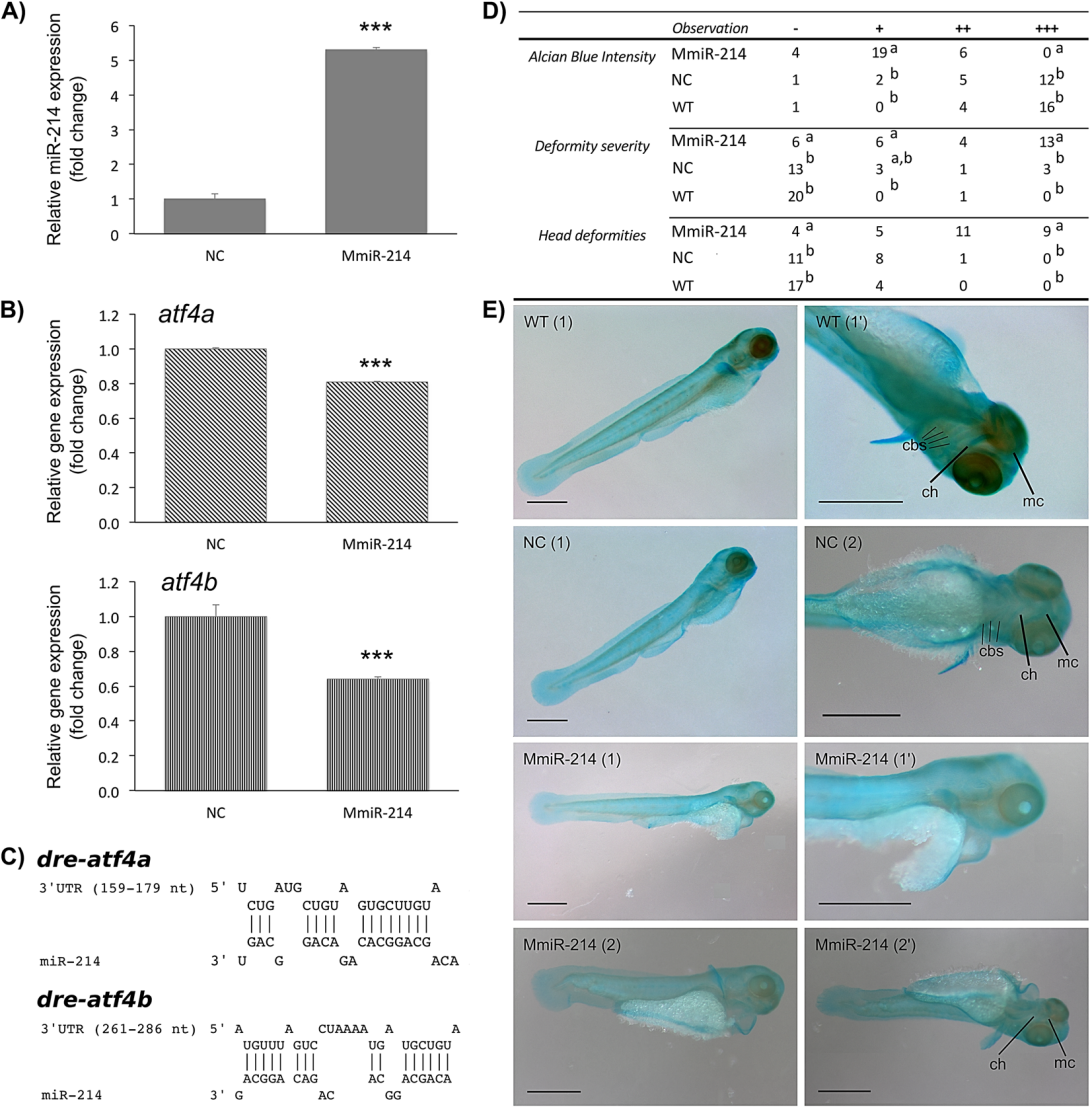
miR-214 ectopic expression downregulates *atf4* transcripts and alters cranial cartilages of zebrafish. Larvae were microinjected at 1-cell stage with MmiR-214 or NC (18 μM) and analysed at 3 dpf. (**A**) Expression of miR-214 was determined by miRNA qPCR and normalized using U6 small RNA. (**B**) Expression of *atf4a and atf4b* in zebrafish larvae microinjected with MmiR-214 or NC. Levels of *atf4a and atf4b* transcripts expressions were determined by qPCR and normalized using 18 S ribosomal RNA housekeeping gene (similar results were obtained using *ef1α* housekeeping gene; data not shown). Results are presented as fold change over NC. Asterisks indicate values statistically different from NC (data are the mean ± s.d. of at least 3 independent replicates; Student’s t-test, ***p < 0.001). (**C**) Predicted miR-214 binding sites in zebrafish *atf4a* and *atf4b* 3′ UTRs. Zebrafish *atf4a* and *atf4b* transcripts sequences were collected from NCBI database and analysed using RNAhybrid. (**D**) Morphological alterations observed on 3 dpf embryos injected with MmiR-214 (n = 29) or NC (n = 21), and wild type (WT; n = 21), stained with alcian blue. Alcian blue intensity was classified as very weak (−), weak (+), normal (++) or intense (+++), while deformities present in the embryos were classified as absent/normal fish (−), low severity (+), severe (++), extremely severe (+++). Different letters indicate statistically significant differences between MmiR-214, NC and WT within the same classification (Chi-square test, p < 0.05). (**E**) Phenotype alterations observed in miR-214-injected embryos comparing to WT and NC-injected embryos, stained with alcian blue. Arrowheads indicate head deformations resulting from flattening of the mandible; arrows indicate differences on staining intensities of main cartilaginous structures (either absent or weak in miR-214-injected embryos). *cbs*, cerathobranchial cartilages; *ch*, ceratohyal; mc, Meckel’s cartilage. (1) or (2) indicate different embryos; (‘) indicate magnifications of the same embryo. Scale bar is 0.5 mm.

In order to confirm the chondrogenic role of miR-214 *in vivo*, and since our previous data revealed that miR-214 was poorly expressed or absent in early stages of zebrafish development (Fig. 1A), we decided to overexpress this miRNA in zebrafish embryos by injecting 1-cell stage zebrafish eggs with miR-214 mimic or negative control (NC). Embryos were analysed at 3 dpf, at the onset of zebrafish craniofacial skeleton formation and when skeletal structures are mainly composed by cartilage^22,23^. For all groups (MmiR-214, NC and WT), the highest mortality was observed at 24 hpf, although miR-214-injected embryos presented twice as much mortality (approximately 30%) comparing to NC (approximately 17%) and WT (approximately 12%). In subsequent days (2 and 3 dpf), mortality decreased drastically in all groups (0–1%). Embryos injected with MmiR-214 had a 5-fold miR-214 up-regulation over NC at 3 dpf (Fig. 5A). Since in previous studies, Atf4 seemed to be pivotal for miR-214 mechanism of action in the mammalian skeleton, we sought to investigate *atf4* expression upon miR-214 ectopic expression. Importantly, we found that both *aft4* paralogs in zebrafish, *atf4a* and *atf4b*, were decreased upon miR-214 overexpression (Fig. 5B). This was consistent with the detection of putative binding sites for miR-214 in both transcripts (Fig. 5C), and further supported the hypothesis that both genes are miR-214 targets in zebrafish. Gross morphology analysis revealed four phenotypes in miR-214-injected embryos comparing to NC: i) the embryos were generally smaller; ii) pericardium was enlarged; iii) the eyes were smaller and less developed; and iv) larvae presented alterations in the size and number of otoliths. For the analysis of structural deformities in cartilage, the embryos were stained with alcian blue, which marks the proteoglycans present in the cartilaginous matrix produced by chondrocytes^45^. This analysis revealed different levels of phenotype severity, which could be due to distinct levels of miR-214 mimic incorporation in each embryo. While almost half of the embryos had severe deformities in the whole body (head and trunk), two thirds presented a clear flattening of the mandible (Fig. 5D,E), a predominant observation in the miR-214-injected embryos. Some embryos also presented a shortening of the trunk due to deformities in the notochord (Fig. 5E). Notably, all miR-214-injected embryos (n = 29) presented a reduced intensity of alcian blue staining indicating that the composition of the matrix in cartilaginous structures was altered and suggesting a defective chondrocyte function (Fig. 5D,E).

**Figure 6:**
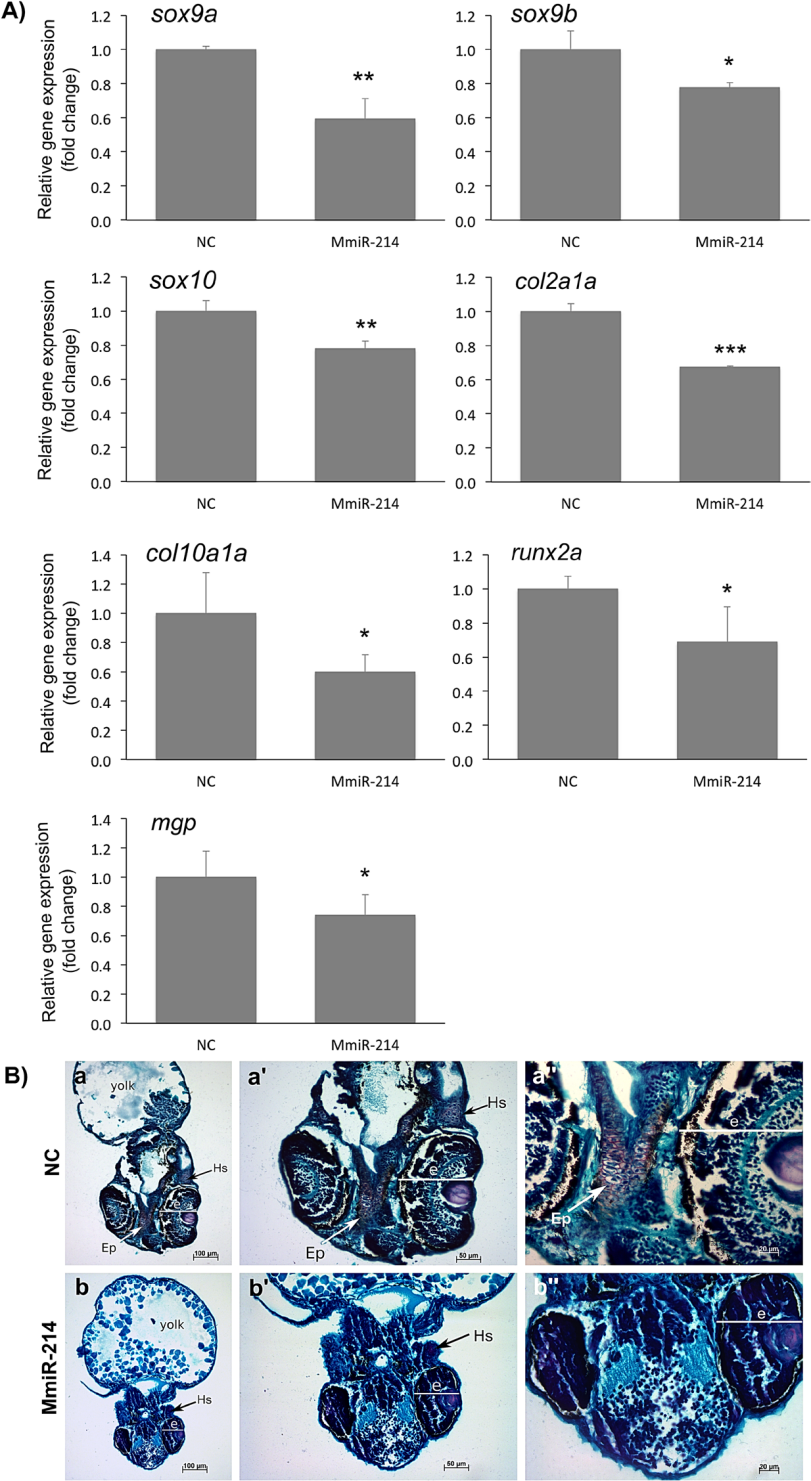
miR-214 ectopic expression downregulates chondrogenic markers and impairs cranial cartilage formation in zebrafish. (**A**) Expression of marker genes of chondrogenesis. Expression levels of *sox9a*, *sox9b*, *sox10*, *col2a1a*, *col10a1a*, *runx2a* and *mgp* were evaluated by standard qPCR of 3 dpf zebrafish embryos microinjected with MmiR-214 or NC (18 μM), and normalized using 18 S ribosomal RNA housekeeping gene (similar results were obtained using *ef1α* housekeeping gene; data not shown). Results are presented as fold change over NC. Asterisks indicate values statistically different from NC (data are the mean ± s.d. of at least 3 independent replicates; one-way Anova, ***p < 0.001; **p < 0.01, *p < 0.05). (**B**) Histological characterization of miR-214 overexpression on zebrafish cartilage formation. 3 dpf embryos microinjected with NC (a) or MmiR-214 (b) were embedded in paraffin, sectioned and stained with the safranin-O/fast green/Mayer’s haematoxylin. Zebrafish eyes (e) are generally underdeveloped in miR-214-injected embryos when compared to NC. Cartilage associated proteoglycans (evidenced by safranin-O) are either absent or present in low amounts in the Ethmoid plate (Ep) or hyosympletic (Hs), respectively, of miR-214-injected embryos comparing to NC. Sections are in the coronal plane; (‘) and (‘’) indicate magnifications of the same section.

In order to get further insight into the effect of miR-214 overexpression in these larvae, we performed gene expression analysis for key markers of cartilage formation: *sox9*, *sox10*, *col2a1*, *col10a1*, *runx2* and *mgp*. Notably, all markers were down-regulated in miR-214-injected embryos (Fig. 6A) demonstrating that in fact cartilage development was hampered. The early marker for cartilage formation, Sox9, is known to be important for neural crest cells commitment to the chondrogenic lineage^46^, morphogenesis and differentiation of cartilage^47^, chondrocyte stacking (*sox9a*) and cell number (*sox9b*)^48^ and chondrocyte hypertrophy^49^. As a transcription factor, Sox9 also regulates the expression of genes crucial for cartilage development, such as Col2a1, Col10a1, Runx2 and Sox10^46,48–51^. Thus, one cannot exclude that the repression of these genes in our experiment could be mediated by *sox9* genes down-regulation itself. Nevertheless, these genes play crucial roles on cartilage development and maintenance^51,52^ and their down-regulation in miR-214-injected embryos further supports a cartilage hindered phenotype. Finally, to further confirm our previous observations, we performed a histological analysis of six miR-214-injected embryos using safranin-O/fast green/Mayer’s haematoxylin staining, to evaluate putative effects on the deposition of proteoglycans in the cartilage matrix. Gross morphological changes mentioned earlier, regarding embryos size, pericardium enlargement and underdevelopment of the eyes, were further confirmed through this analysis (Fig. 6B and Supplementary Fig. S7). Notably, five out of the six analysed embryos exhibited an almost complete absence of safranin-O staining (Fig. 6B and Supplementary Fig. S7) indicating a severe loss of reactivity with proteoglycans, and further suggesting that the cartilaginous matrix is altered and cartilage formation is impaired. Noteworthy, in all miR-214-injected embryos, the ethmoid plate, a cartilaginous structure considered a model for the mammalian palate formation^53^, was absent (Fig. 6B). Moreover, the few cells reactive to safranin-O in the hyosympletic of miR-214-injected embryos were clearly delayed in differentiation and lacked an organized arrangement when compared to the NC embryos (Fig. 6B). In fact, we could not detect the presence of mature chondrocytes in miR-214-injected embryos as evidenced in NC (Fig. 6B and Supplementary Fig. S7). Nevertheless, some cartilaginous structures, e.g. pharyngeal arch cartilage (Supplementary Fig. S7A and B), seemed to be properly placed and resembled a chondrocyte packing, but lacked reactivity to safranin-O in the miR-214-injected embryos. Other structures, e.g. pectoral fins, were present in the miR-214-injected embryos, but their cells were apparently less differentiated compared to NC, and lacked safranin-O staining (Supplementary Fig. S7C and D). In general, our histological analysis confirmed that chondrogenesis is impaired when miR-214 is overexpressed, and suggested that chondrocytes lost their ability to differentiate and produce the main components of cartilage ECM. This result is consistent with the previously observed down-regulation in the levels of expression of markers of cartilage formation. Altogether, data collected in this report pointed towards a putative negative role of miR-214 on vertebrate cartilage formation.

## Conclusions

**Figure 7:**
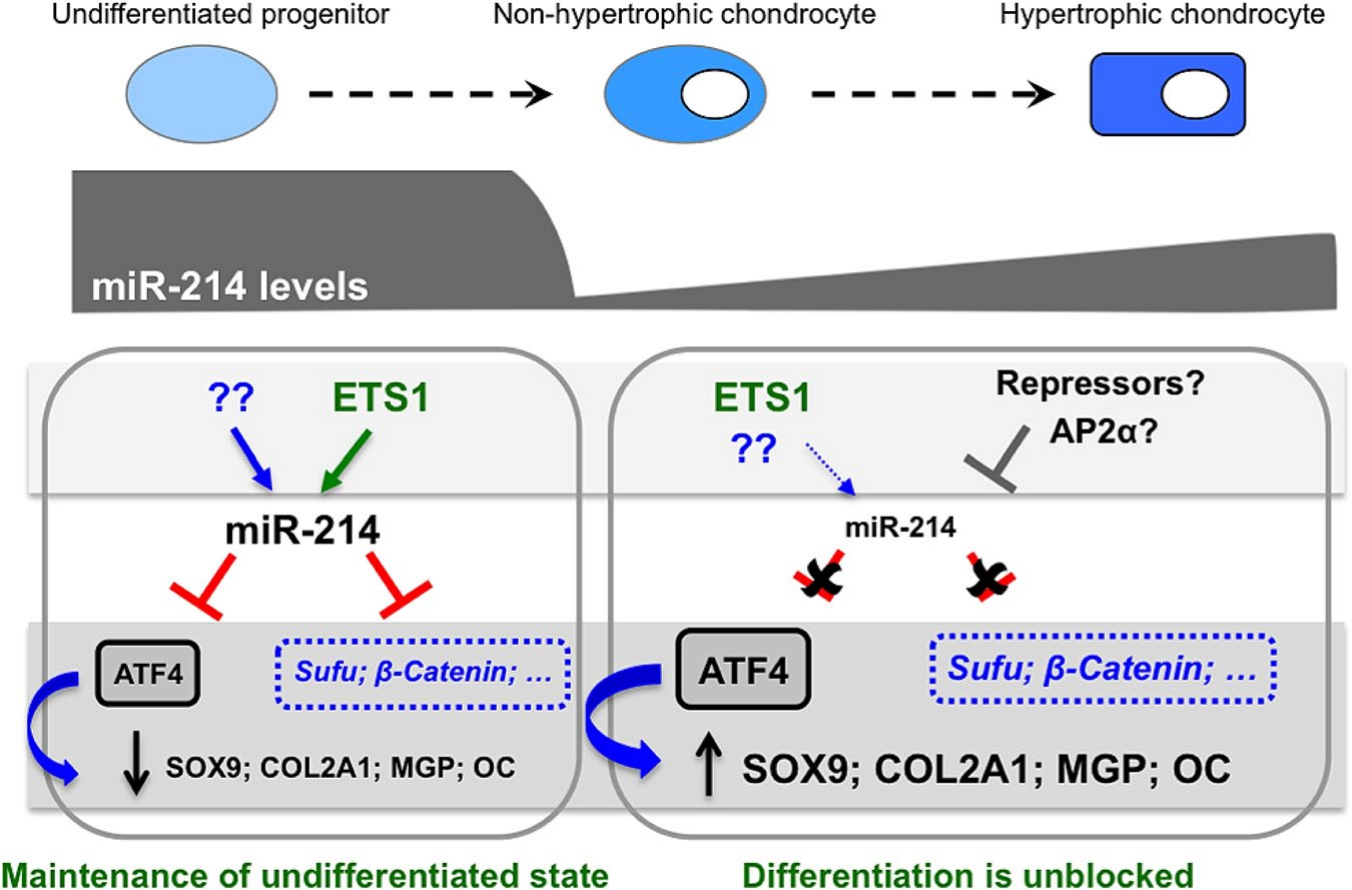
A model for miR-214 mode of action in chondrogenesis. The levels of miR-214 during chondrogenesis are distinct in undifferentiated cells and in differentiating cells. ETS1 promotes miR-214 expression in undifferentiated cells, although other factors might impact on miR-214 expression. During differentiation, transcriptional repressors such as AP2α should overcome ETS1 action leading to the repression of miR-214 transcription and downregulate its expression. Higher levels of miR-214 contribute to maintain an undifferentiated cell state, possibly by blocking ATF4 and/or by controling Hh and WNT pathways through targeting of Sufu and β-Catenin, respectively. These actions impact on the expression of genes necessary for chondrocyte differentiation (such as SOX9 and COL2A1), which are maintained at basal levels. For chondrocyte differentiation to progress, miR-214 levels decrease thus alleviating the expression of their targets as well as the expression of chondrocyte markers. Arrows indicate activation and their relative size indicates an incremented effect. Green arrow indicates the transcriptional activation identified in this study; grey intersected line indicates transcriptional repression; red intersected lines indicate repression by miRNA; dashed black arrows indicate differentiation processes. Genes in blue letters, blue intersected lines and blue arrows represent proposed mechanisms not yet, or not completely, demonstrated.

Herein, we show that miR-214 has a role in chondrogenesis and an underlying mechanism for miR-214 action is proposed. The pattern of expression of miR-214 during zebrafish development and ATDC5 chondrogenic differentiation suggested that this miRNA was most likely associated to chondrogenesis. This was evidenced by the high levels of miR-214 expression found during early stages of cartilage formation in zebrafish and in undifferentiated ATDC5 cells. Since miR-214 expression strongly decreased upon ATDC5 cell differentiation, we speculated if miR-214 could be a natural inhibitor of chondrogenesis. In fact, *in vitro* and *in vivo* gain-of-function experiments revealed that miR-214 was able to inhibit chondrogenesis, as evidenced by down-regulation of chondrogenic marker genes, and by the observation of phenotypic changes in cartilage formation in zebrafish miR-214-injected embryos. Loss-of-function experiments further supported that miR-214 levels should be important for proper chondrocyte differentiation, although it also revealed that the role of miR-214 might be more complex than originally expected. We propose that at least part of miR-214 effects in chondrogenesis are Atf4-dependent since this gene was consistently decreased upon miR-214 overexpression and unaltered upon miR-214 down-regulation, thus suggesting a direct repressive effect by miR-214 (Fig. 7), as previously validated by other authors^10^. Interestingly, miR-214 overexpression lead to an overall down-regulation of Atf4 putative transcriptional targets such as *Sox9*, *Col2a1* and *Mgp*, where we have identified several conserved putative-binding sites for Atf4, and also for Runx2, one of its skeletal cooperative partners (Supplementary Fig. S8). In addition, reporter assays testing the promoter of one of these genes, the MGP human promoter, evidenced that co-expression (through co-transfection experiments in ATDC5 cells) of Atf4 with its TF partners, Runx2 and Satb2, was able to enhance its promoter activity in ATDC5 cells (Supplementary Fig. S8). Even though, one must consider that miR-214 is also known to control signalling pathways crucial for chondrogenesis, such as the Hh and WNT pathways by targeting Sufu and β-Catenin respectively^19,44^. These putative regulations might also contribute for miR-214 mode of action.

Based on our results, we propose that low levels of miR-214 are most likely required in order for chondrogenesis to initiate/proceed. High levels of miR-214 during early chondrocytic cell differentiation seem to delay this process by affecting the expression of important players of chondrogenesis, as Sox9. miR-214 mode of action could be in part explained by direct repression of *Atf4*. Once miR-214 expression decreases, Atf4 levels also increase, and chondrocytic differentiation proceeds (Fig. 7). Taken together, our results evidence for the first time that miR-214 could have an important role on chondrogenesis and cartilage formation, similarly to what was previously observed in osteogenesis.

## Material and Methods

### Ethics Statement

All operators involved in animal handling and experimentation are legally accredited by the Portuguese Direção Geral de Alimentação e Veterinária (DGAV) and all the experimental procedures involving animals followed the EU (Directive 86/609/CEE) and National (Portaria no. 1005/92 de 23 de Outubro; Portaria no. 466/95 de 17 de Maio; Portaria no. 1131/97 de 7 de Novembro) legislation for animal experimentation and welfare. Animal experimental procedures were performed under the authorization 0421/000/000 from the DGAV complying with the decreto de lei 113/2013 de 7 de Agosto, from the Portuguese legislation.

### Biological samples

Zebrafish eggs were obtained from natural spawning of a wild-type (AB) broodstock, and fish and larvae were maintained and raised using standard methods^54^. For total RNA, pools of up to twenty zebrafish embryos, larvae and juvenile were collected at 1 k-cell (approximately 3 hours post fertilization, *hpf*), 18 somites (approximately 16 hpf), 24, 36 hpf, 2, 4, 6, 15, 30, 45, 60 and 81 days post fertilization (*dpf*), and from adult male and female. RNA was also isolated from different adult tissues of zebrafish specimens (muscle, branchial arches, skull and vertebra) and mice specimens (muscle, ear, femur, calvaria and vertebra). Animals were euthanized before sampling with 600 ppm MS222 (Sigma-Aldrich) for zebrafish and anesthesia followed by cervical dislocation for mouse.

### RNA extraction, reverse-transcription and Real-time quantitative PCR (qPCR)

Samples were collected in TRI Reagent (Sigma-Aldrich) (10 volumes/weight) and the total RNA extracted according to manufacturer’s protocol. For expression analysis of miRNAs, 1 μg of total RNA was polyadenylated, reverse-transcribed and amplified using miRNA-specific primers (Supplementary Table S1) using the NCode miRNA First-Strand cDNA Synthesis and NCode SYBR miRNA qPCR kits (Invitrogen), according to manufacturer’s instructions. For gene expression analysis, 1 μg of total RNA was reverse transcribed using MMLV-RT (Invitrogen), and transcripts amplified with gene-specific primers (Supplementary Table S1) and SsoFast EvaGreen supermix (Bio-Rad), according to manufacturer’s instructions. qPCR was performed using the StepOnePlus system (Applied Biosystems), unless stated otherwise, and relative gene expression was calculated using the ΔΔCt method^55^. Expression of miRNAs was normalized using the expression levels of U6 small nuclear RNA (U6), while mRNAs expression was normalized using expression levels of hypoxanthine phosphoribosyltransferase 1 (*Hprt1*) for mice samples and 18 S ribosomal RNA housekeeping gene for zebrafish samples.

### *In Situ* Hybridization (*ISH*)

Zebrafish larvae and juveniles at 10, 20 and 90 dpf were euthanized with a lethal dose of MS222 (Sigma-Aldrich) and fixed for 24 hours (h) in 4% paraformaldehyde (PFA) at 4 °C. Specimens were further decalcified in a 10% ethylenediaminetetraacetic acid (EDTA)/2% PFA solution for a minimum of 2 weeks and up to 2 months depending on their size. Samples were then washed in phosphate buffered saline (PBS) and maintained in 100% methanol at −20 °C until processing. For ISH, specimens were embedded in paraffin and sectioned (4–6 μm thick). Detection of dre-miR-214 was performed using an ISH protocol adapted from the method described by Kloosterman *et al*.^56^ using LNA (Locked Nucleic Acid)-modified oligonucleotide 5′-Digoxigenin (DIG) labelled probe. Briefly, sections were fixed in 4% PFA and treated with 10, 20 or 40 μg/ml of proteinase K for specimens with 10, 20 and 90 dpf, respectively. After 2 h in pre-hybridization solution (50% formamide, 5 × saline sodium citrate buffer (SSC), 500 μg tRNA, 50 μg Heparin, 0.1%Tween and 9.2 mM of citric acid), sections were incubated with 40 nM of LNA ISH probe (Exiqon) specific for detection of dre-miR-214 (Supplementary Table S1). As negative control, sections were hybridized with a scramble probe (Supplementary Table S1). After 16 h incubation at 55 °C in a humidified chamber (5 × SSC), sections were washed with decreasing concentrations of formamide/SSC, then with PBST (PBS with 0.1% Tween-20) and incubated again for 1 h with blocking buffer (2% blocking solution from Roche diluted in maleic acid, 2% (v/v) sheep serum and 2% (m/v) bovine serum albumin (BSA)). Anti-DIG antibody conjugated with alkaline phosphatase (Roche, diluted 1:1600) was added to each section and incubated for 16 h at 4 °C. Each section was washed 5 times with PBST, 3 times with AP buffer (100 mM Tris, 100 mM NaCl, 50 mM MgCl_2_, pH 9.5) and incubated with 75 mg/mL NBT/ 50 mg/mL BCIP in AP buffer for signal detection. Sections were air-dried, mounted with Eukitt (Sigma-Aldrich) and imaged by microscopy (Olympus IX-81 inverted microscope).

### Identification and analysis of *Dnm3os* promoter sequences

Pre-miR-199a-2 sequences were retrieved from miRbase database (http://www.mirbase.org/) for: *Homo sapiens*, *Mus musculus*, *Xenopus tropicalis*, *Takifugu rubripes*, *Oryzia latipes*, *Gasterosteus aculeatus*, *Tetraodon nigroviridis* and *Danio rerio*, blasted at Ensembl genome browser (www.ensembl.org) and genomic sequences with 2.5 kilobase (Kb) were collected starting immediately upstream of pre-miR-199a-2 (now on called *Dnm3os* promoter). Multiple sequence alignment of *Dnm3os* promoter was performed using CHAOS/DIALIGN (http://dialign.gobics.de/chaos-dialign-submission) and fed to ConTra v2 (http://www.dmbr.ugent.be/prx/bioit2-public/contrav2/) for search of putative conserved TFBS. Stringency parameters: core match = 0.95 and similarity matrix = 0.85. The human, mouse and zebrafish promoter sequences were also fed to MatInspector (Genomatix Software GmbH) and only the TF predicted by the two algorithms were considered. Additionally, we analysed Chromatin Immunoprecipitation studies where some of the TFBS here predicted were found to physically interact with DNM3OS promoter, available through Genome Browser at UCSC (http://genome.ucsc.edu/), thus supporting our in silico analysis.

### Cloning of zebrafish *dnm3os* 5′ end

The 5′ end of zebrafish *dnm3os* was achieved by rapid amplification of cDNA ends (RACE) using Advantage cDNA polymerase mix (Clontech) and a zebrafish Marathon cDNA library as template previously prepared^57^, according to manufacturer’s instructions (Clontech). Specific reverse primers (Supplementary Table S1) were designed based on the dre-pre-miR-199a-2 sequence available in miRbase (http://www.mirbase.org/) and combined with universal adapter primers (AP1 and AP2 universal primers; Supplementary Table S1). Amplified PCR products were subsequently inserted into pCRII-TOPO (Invitrogen) and further analysed by standard DNA sequencing.

### Plasmid Constructs

Zebrafish (2.3 kb) and human (2.4 kb) *Dnm3os* promoter fragments were amplified using specific primers (Dre PR Fw and Dre PR Rev, Hsa PR Fw and Hsa PR Rev, in Supplementary Table S1) and genomic DNA as template. PCR products were cloned into pCRII-TOPO vector (Invitrogen), confirmed by DNA sequencing, and further used as templates to amplify new cDNA fragments containing specific deletions of the promoter of each species. Forward and reverse primers used to amplify these fragments contained 5′ ends with *Nhe*I and *Bgl*II restriction site sequences, respectively, which were used for cloning into pGL3-Basic luciferase reporter gene vector (Promega). Constructs generated in pGL3-basic were as follow: full, partial and TATA less zebrafish promoters (−1926bp/+ 244 bp, −657bp/+ 244 bp and −1926bp/−140bp, respectively) and full, partial and TATA less human promoters (−2299bp/+ 190 bp, −641bp/+ 190 bp and −2299bp/−30bp, respectively).

The pCMX-ETS1 and pCMX-TWIST1 constructs were obtained by cloning cDNA fragments of zebrafish open reading frame (ORF) of v-ets avian erythroblastosis virus E26 oncogene homolog 1 (*ets1*) (nucleotides 129–1427, GenBank accession KF774190) and *twist1a* (nucleotides 114–629, GenBank accession NM_130984.2), from 48 hpf larvae and 69 dpf juveniles respectively, into pCMX-PL2 expression vector (kindly provided by Dr. Roland Schüle, Universitats-Frauenklinik, Klinikum der Universitat Freiburg, Freiburg, Germany). *Bam*HI and *Nhe*I restriction site sequences were incorporated into the forward and reverse primers, respectively, and the resulting PCR products were digested and cloned into the corresponding sites in pCMX-PL2 vector. Plasmids used to express TFs in mammalian systems were kindly provided by Dr Joseph P. Stains^58^ University of Maryland, School of Medicine, Baltimore, MD, for pcDNA3-SP1 and pcDNA3 control plasmid, from Dr Ann Ehrlund^59^ Karolinska Institutet, Department of Medicine, Huddinge, Stockholm for pcDNA3.1-TWIST1 and from Dr José Bragança, CBME, University of Algarve^60^ for pcDNA3.1-AP2α. The identity of all constructs was confirmed by DNA sequence analysis.

### Cell culture

ABSa15 cells were cultured in DMEM supplemented with 10% fetal bovine serum (FBS) and 0.2% fungizone, and incubated at 33 °C in 10% CO_2_. MC3T3-E1 and ATDC5 cells were cultured in α-MEM (supplemented with 10% FBS) and DMEM:F12 (supplemented with 5% FBS) respectively, and incubated at 37 °C in 5% CO_2_. Cells were sub-cultured every 2–3 days by trypsinization. All culture media were supplemented with 1% penicillin/streptomycin and 2 mM L-Glutamine. Cell culture media and FBS were obtained from Sigma-Aldrich and all other supplements and antibiotics were obtained from Gibco.

### Dual-Luciferase Reporter Assays

Cells were seeded in 12-well plates, further cultured for 14–16 h, transfected with X-tremeGENE HP (Roche) according to manufacturer’s instructions, and as follows: ABSa15 (8 × 10^4^ cells/well) with 500 ng of luciferase construct and 300 ng of pRL-SV40 vector (Promega), ATDC5 (1 × 10^5^ cells/well) with 500 ng of luciferase constructs and 50 ng of pRL-null vector and MC3T3 (8 × 10^4^ cells/well) with 500 ng of luciferase construct and 200 ng of pRL-null vector. For co-transfections 50 ng of each TF construct or empty vector (control) was added to the previous conditions. 48 h after transfection, cells were lysed and luciferase activities were measured using Dual-Luciferase Reporter Assay system (Promega) in a Synergy 4 microplate reader (Biotek). Relative luciferase activity was determined as the ratio of firefly/renilla (F-Luc/R-Luc).

### Chromatin Immunoprecipitation

ChIP assays were performed as previously described^61^ with minor modifications. Briefly, ATDC5 cells were seeded in 145 mm petri dish (Greiner; 1,2 × 10^7^ cells per dish) and incubated for 24 hours at 37 °C and 5% CO_2_. After cross-linking with formaldehyde, chromatin from ATDC5 cells was immunoprecipitated with specific antibodies against TWIST1, ETS1, SP1 (H-81 × , C-20 × and E-3 × respectively, Santa Cruz Biotechnology INC.)^62–64^, RNA polymerase II (ab5408, Abcam)^65^ anti-mouse IgG antibody (whole anti-serum, Sigma-Aldrich). The recovered DNA was analysed by qPCR in an ABI7300 sequence detection system (Applied Biosystems) with SYBR green Master Mix (Fermentas), using primers covering the proximal promoter region of the *Dnm3os* gene (Supplementary Table S1).

### miR-214 overexpression during ATDC5 cell differentiation

Cells were seeded in 24-well plates (2.5 × 10^4^ cells/well), incubated for 16 h and transfected with miRIDIAN microRNA mimic for mmu-miR-214 (from now on designated MmiR-214) or negative control scrambled miR 1 (NC) (Dharmacon) at a final concentration of 50 nM, using EzWay (Koma Biotech) transfection reagent. Then, cells were grown until confluence (T0) and differentiation was induced by supplementing medium with ITS mixture (10 μg/mL Insulin, 5.5 μg/ml transferring, 6.7 ng/ml sodium selenite, Gibco) and replaced every 2–3 days. A second transfection was performed 10 days after the first one, using the same procedure. At appropriate times, cells were collected for total RNA extraction.

### Microinjection of zebrafish and *in vivo* effect of miR-214 overexpression

One-cell stage embryos were microinjected with 4,6 nl of MmiR-214 or NC at 18 μM in 1 × Danieau solution (58 mM NaCl, 0.7 mM KCl, 0.4 mM MgSO4, 0.6 mM Ca(NO3)2, 5.0 mM HEPES pH 7.6), under a MZ6.0 stereomicroscope (Leica) using a Nanoliter 2010 microinjector (World Precision Instruments LLC). Embryos were raised at 28.5 °C ± 1 °C until 3 dpf and then anesthetized with a lethal dose (200 ppm) of MS-222 (Sigma-Aldrich). For characterization of cartilaginous structures, specimens were fixed for 2 hours in 4% PFA, and either stained with 0.1% alcian blue 8GX (Sigma-Aldrich) (20 to 30 larvae per group) as described^66^ or embedded in paraffin, sectioned (4–6 μm thick) and stained with safranin O/fast green/mayer’s hematoxylin (6 larvae). Images where aquired in a SteREO Lumar.V12 stereoscope or in a AxioImager Z2 microscope (Carl Zeiss). For gene expression analysis, 25 larvae per group were collected, and expression of miRNA or mRNAs was performed using gene-specific primers (Supplementary Table S1) and the CFX96^TM^ Real-Time System (Bio-Rad). Analysis of putative miR-214 binding sites analysis was performed using RNAhybrid (https://bibiserv2.cebitec.uni-bielefeld.de/rnahybrid) and PITA algorithms (https://genie.weizmann.ac.il/pubs/mir07/mir07_prediction.html). Only binding sites predicted by both databases were considered.

### Statistical analysis

Statistical analysis was performed with GraphPad Prism 5 (GraphPad). Comparisons between two groups were made using a two-tailed unpaired Student’s t-test. For comparisons between multiple groups, one-way ANOVA followed by Tukey’s Multiple Comparison Test, was used. Qualitative variables were analysed with Chi-Square Test. Differences were considered statistically significant for p < 0.05.

## Electronic supplementary material

Supplementary Information
