# Supplementary material for: Evidences for a New Role of miR-214 in Chondrogenesis
Source: Sci Rep. 2018 Feb 27;8:3704. doi: 10.1038/s41598-018-21735-w (PMC5829070; doi:10.1038/s41598-018-21735-w)
Supplement: Supplementary file 1 — Supplementary Information [file 41598_2018_21735_MOESM1_ESM.pdf]

Evidences for a New Role of miR-214 in Chondrogenesis

Vânia Palma Roberto[1,2], Paulo Gavaia[1,3], Maria João Nunes[4], Elsa Rodrigues[4,5], Maria Leonor Cancela[1,3*] and Daniel Martins Tiago[1*]

[1]Centre of Marine Sciences (CCMAR/CIMAR-LA), University of Algarve, 8005-139 Faro, Portugal

[2]PhD Program in Biomedical Sciences, DCBM, University of Algarve, 8005-139 Faro, Portugal.

[3]Department of Biomedical Sciences and Medicine, University of Algarve, 8005-139 Faro, Portugal.

[4]Instituto de Investigação do Medicamento (iMed.ULisboa), Faculty of Pharmacy, Universidade de Lisboa, Av. Prof. Gama Pinto, 1649-003 Lisbon, Portugal

[5]Department of Biochemistry and Human Biology, Faculty of Pharmacy, Universidade de Lisboa, Av. Prof. Gama Pinto, 1649-003 Lisbon, Portugal

***Corresponding authors:** Daniel M. Tiago (dtiago@ualg.pt) and M. Leonor Cancela (lcancela@ualg.pt)

SUPPLEMENTARY INFORMATION

1) Supplementary Figures

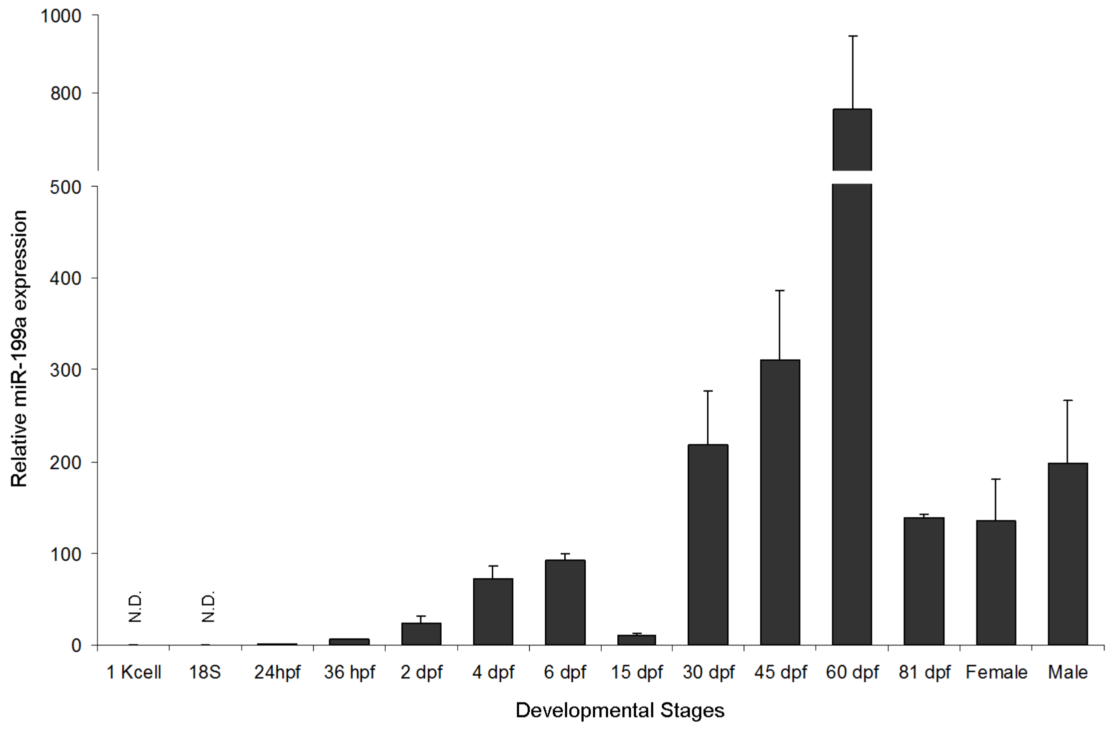

Supplementary Figure S1. Relative expression of miR-199 during developmental stages of zebrafish. Levels of miR-199 expression were measured by miRNA specific qPCR analysis, using total RNA samples from different stages of zebrafish development, and normalized using zebrafish U6 small RNA and 24 hpf as reference sample. Values are the mean ± s.d. of at least 3 independent replicates; *hpf*, hours post fertilization, *dpf*, days post fertilization.

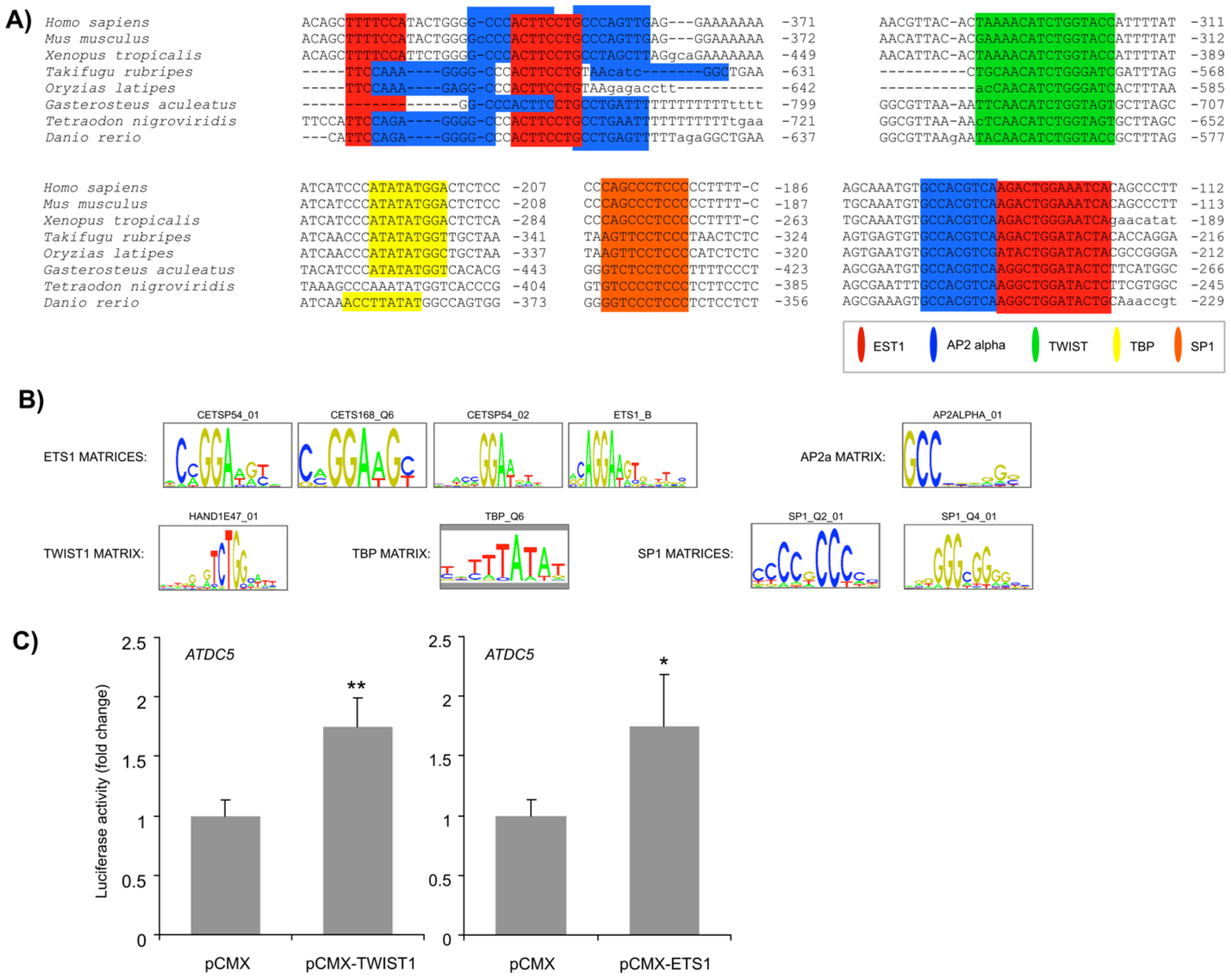

Supplementary Figure S2. Analysis of *Dnm3os* promoter transcriptional regulation. For identification and localization of conserved TF binding sites, *Dnm3os* putative promoter sequences of *Homo sapiens*, *Mus musculus*, *Xenopus tropicalis*, *Takifugu rubripes*, *Oryzia latipes*, *Gasterosteus aculeatus*, *Tetraodon nigroviridis* and *Danio rerio* were aligned using CHAOS/DIALIGN, and then fed to ConTra v2 (http://bioit.dmbr.ugent.be/contrav2/) to search for conserved TFBS; the following parameters were used: core match=0.95, similarity matrix=0.85, TRANSFAC database. **A)** Conserved putative binding sites for ETS1, AP2alpha, TWIST1, TBP and SP1 are shown. Number of the last nucleotide shown in the alignment is displayed at the right, considering that -1 nt is the first nucleotide upstream of pre-miR-199a. **B)** Sequence logos of positional weight matrices for EST1, AP2alpha, TWIST1, TBP and SP1, as provided by ConTra v2. For TBP, parameters were: core match=0.90, similarity matrix=0.75. **(C)** For TF effect on zebrafish *Dnm3os* promoter, ATDC5 cells were co-transfected with 500 ng/well of full promoter construct in 12-well plates, and 50 ng/well of either pCMX-TWIST1 or pCMX-ETS1**,** or empty vector as control. Renilla and firefly luciferase activities were determined 48 h after transfection. Results are indicated as fold change over the control empty vector pCMX-PL2. Data are the mean ± s.d. of at least 5 independent experiments (Student's t-test, *p<0.05, **p<0.01).

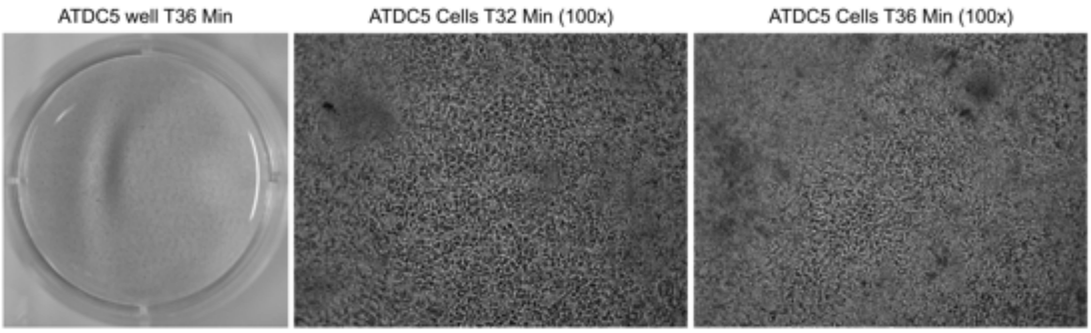

Supplementary Figure S3. ATDC5 cells extracellular matrix mineralization. Mineralization nodules were assessed by von Kossa staining at T32 and T36. Mineral deposition was revealed through von Kossa staining as follows: ATDC5 cells were fixed in 4% PFA at 4ºC for 1h, washed and stained with 5% $AgNO_3$ for 30 min under UV light.

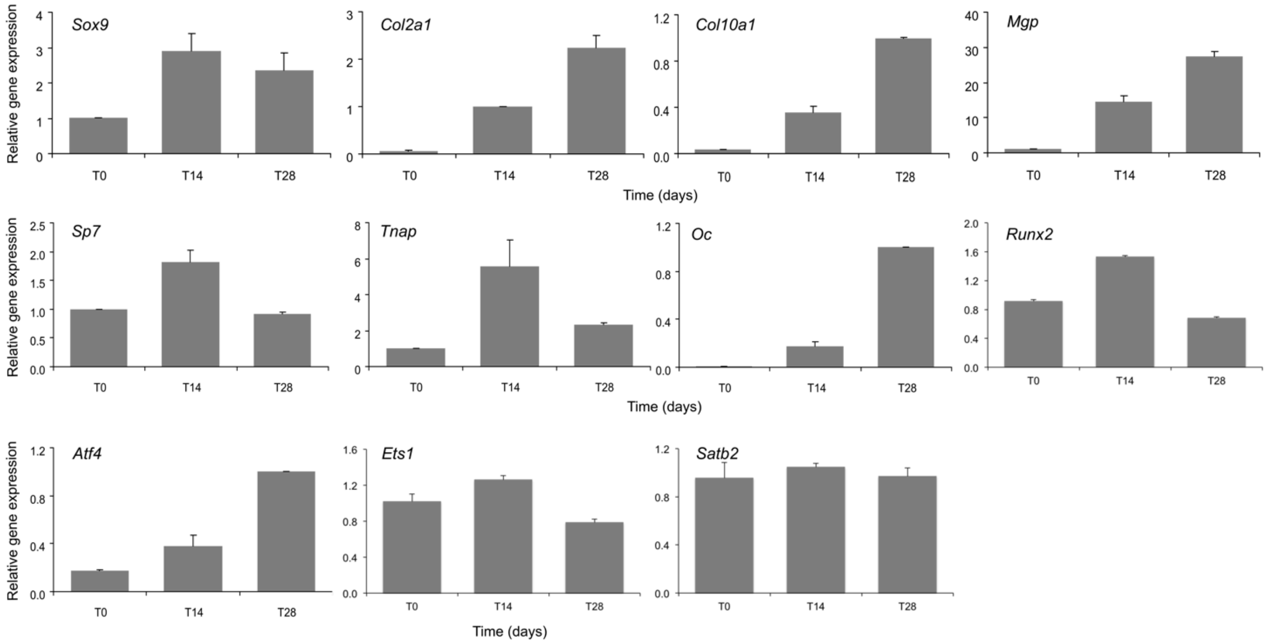

Supplementary Figure S4. Relative gene expression of different genes associated with chondrogenic differentiation in ATDC5 cells. Expression levels of sex determining region Y-box 9 (*Sox9*), type II collagen α1 (*Col2a1*), type X collagen α1 (*Col10a1*), matrix Gla protein (*Mgp*), osterix (*Sp7*), alkaline phosphatase liver/bone/kidney (*Tnap*), osteocalcin (*Oc*), runt related transcription factor 2 (*Runx2*), activating transcription factor 4 (*Atf4*), ETS proto-oncogene 1, transcription factor (*Ets1*) and special AT-rich sequence binding protein 2 (*Satb2*) were evaluated by qPCR analysis of total RNA samples collected from confluent cultures (T0) of ATDC5, and after 14 (T14) and 28 (T28) days of differentiation. Gene expression was normalized using *Hprt1* housekeeping gene expression (similar expression data was collected using *Hprt6* and *Gapdh* housekeeping genes; data not shown). Values are the mean ± s.d of at least 3 independent replicates.

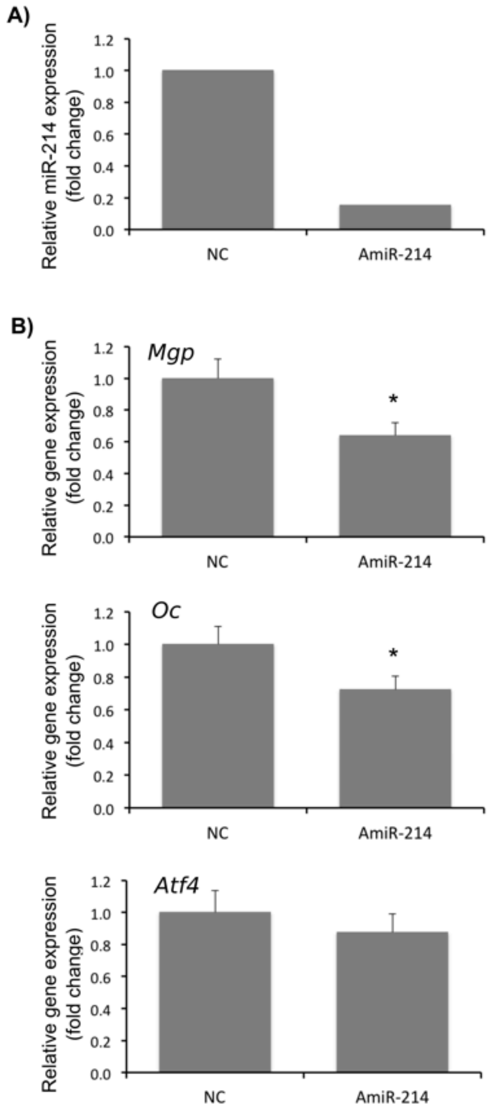

Supplementary Figure S5. Effect of miR-214 knockdown on the levels of marker genes for chondrocyte differentiation in ATDC5 cells. ATDC5 cells were transfected with 50 nM of antagomiR-214 (AmiR-214) or antagomiR-Control (NC), and differentiation was induced at confluence (T0) for 14 days as described for mimic experiments in material and method section. A) Levels of expression of miR-214 were determined by miRNA qPCR analysis and normalized using U6 small RNA. B) Levels of expression of Mgp, Oc and Atf4 were determined by standard qPCR and normalized using Hprt1 housekeeping gene (similar results were obtained using Gapdh housekeeping gene; data not shown). Results are presented as fold change over NC. Asterisks indicate values statistically different from NC (data are the mean ± s.d. of at least 3 independent replicates; Student's t-test, $*p<0.05$).

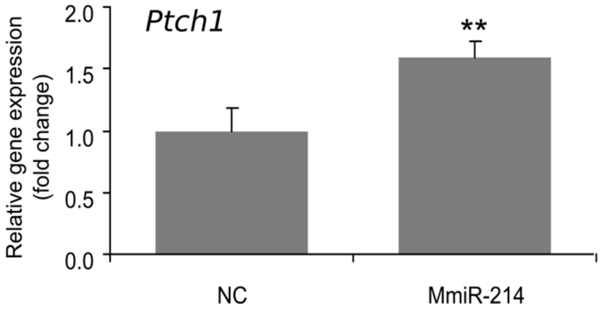

Supplementary Figure S6. Patched 1 (Ptch1) expression in ATDC5 undergoing differentiation and transfected with mmu-miR-214 mimic (MmiR-214) or corresponding negative control (NC). MmiR-214 or NC were transfected into ATDC5 cells 16 hours after seeding and differentiation was induced when cells reached confluence (T0). After 14 days of treatment, collected RNA samples were used to determine the expression of Ptch1 and normalized using HPRT1 housekeeping gene (similar expression data was collected using HPRT6 and GAPDH housekeeping genes; data not shown). Results are presented as fold change over NC. Asterisk indicates value statistically different from NC (Values are the mean of at least 3 independent replicates; Student's t-test, $^{**}p < 0.01$).

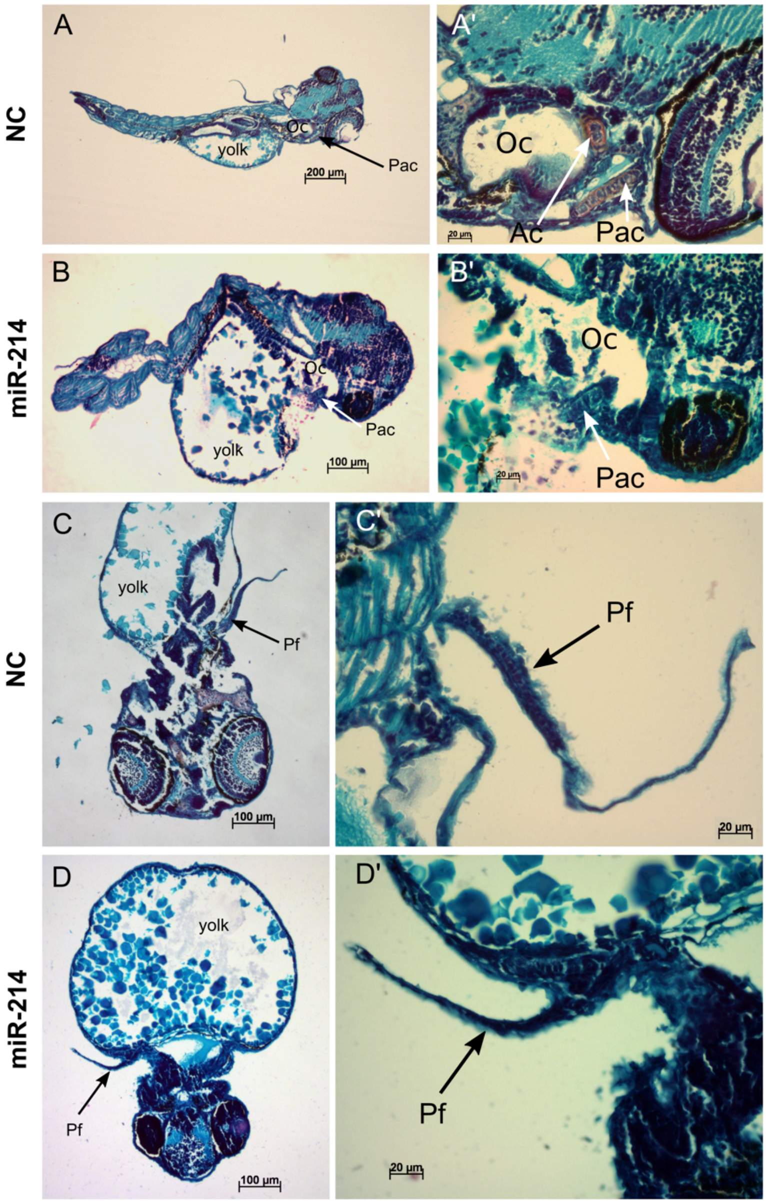

Supplementary Figure S7. Histological characterization of miR-214 effect in zebrafish. Zebrafish embryos (3 dpf) injected with NC (**A** and **C**) or miR-214 mimic (**B** and **D**) were embedded in paraffin, sectioned and stained with the Safranin-O/Fast Green/Mayer's Haematoxylin staining. In NC embryos, pharyngeal arch cartilage (Pac) (arrow in A and A') and chondrocytes of the pectoral fins (Pf) (arrow in C and C') are clearly stained with Safranin-O, but not in miR-214-injected embryos (arrows in B, B', D and D'). *Ac*, auditory capsule; *Pac*, pharyngeal arch cartilage; *Oc*, otic capsule; *Pf*, pectoral fin; (') indicate magnification of the same section.

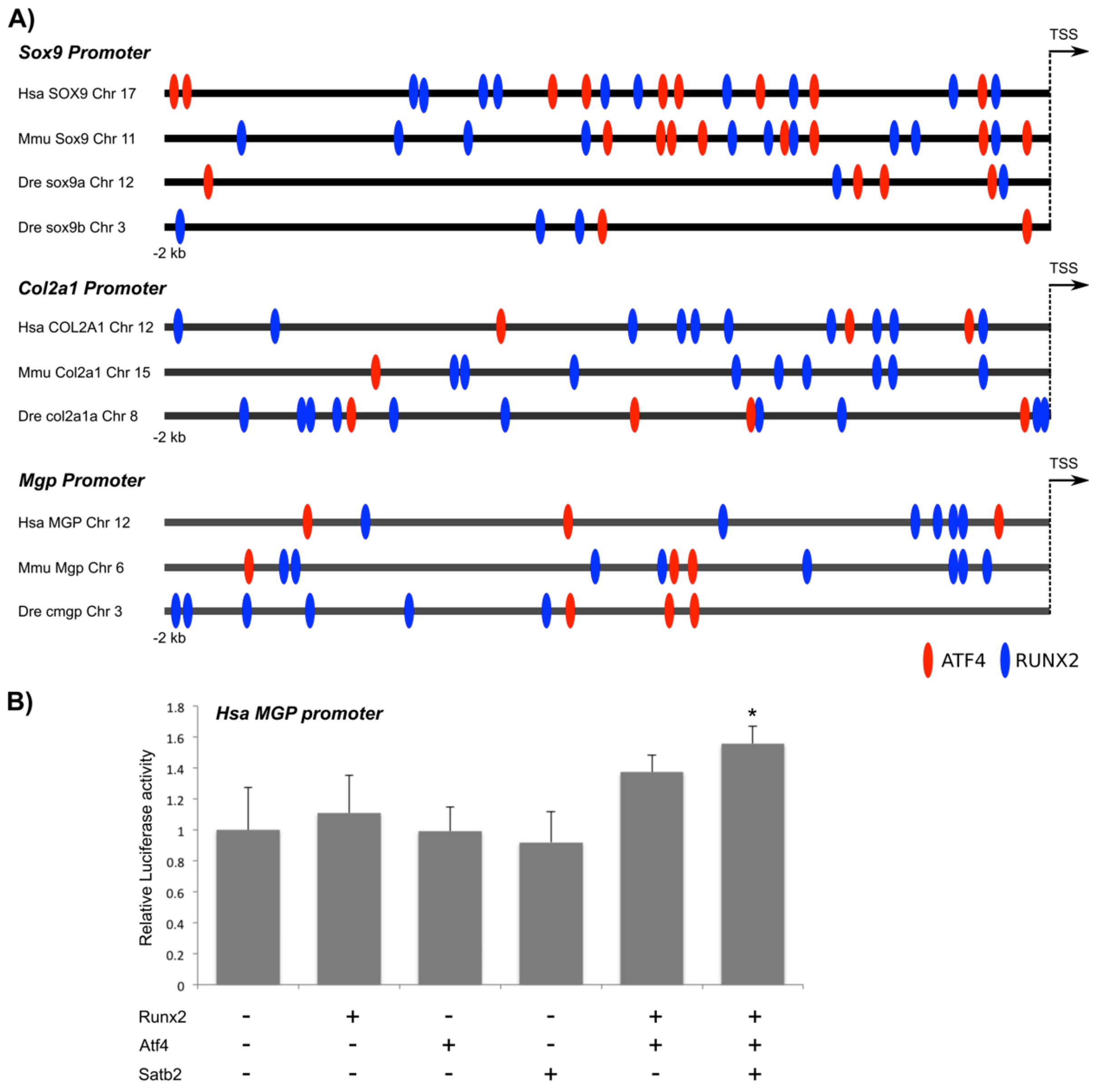

Supplementary Figure S8. Putative transcriptional target genes of Atf4. (A) Schematic representation of *Sox9*, *Col2a1* and *Mgp* putative promoters. Genomic sequences (~ 2 kb) from *Homo sapiens*, *Mus musculus* and *Danio rerio* upstream the transcriptional start site (TSS) of each gene were retrieved from Ensembl, aligned using CHAOS/DIALIGN and fed to ConTra v2 (http://bioit.dmbr.ugent.be/contrav2/) to search for conserved binding sites of both Atf4 and Runx2; the following parameters were used: core match=0.90, similarity matrix=0.75, TRANSFAC database. **(B)** PGL3-Human MGP promoter (3.6 kb, 500 ng/mL) was co-transfected with Runx2, Satb2 and Atf4 (50 ng/mL) alone or in combinations or with negative control vector (PCMX-PL2), into ATDC5 cells. Firely and renilla Luciferase were measured 48 h after transfection. Values are the mean ± s.d of at least 3 independent replicates (One-way Anova, * $p < 0.05$). Atf4 was amplified from RNA of ATDC5 cells and sub-coned into PCMX-PL2; while Runx2, human MGP promoter construct and Satb2 were a kind gift from Dr. Gerard Karsenty (Baylor College of Medicine, Houston, TX, USA), Dr. Roland Schüle (Universitats-Frauenklinik, Klinikum der Universitat Freiburg, Freiburg, Germany) and a Dr. Rudolf Grosschedl (Max-Planck-Institute for Immunobiology and Epigenetics, Freiburg, Germany) respectively.

2) Supplementary Tables

Name	Sequence
**Primers used for cloning of zebrafish Dnm3os 5' UTR**	
Dre Cluster Rev1	5'-CAGGTAGTCTGAACACTGGGATGACG-3'
Dre Cluster Rev2	5'-GATAGTTCCAGCCCTCCCTCTCTCTC-3'
**Primers used for cloning of zebrafish and human Dnm3os promoters**	
Dre PR Fw	5'-CGCATACGGTCTAGTCGCAGGAGTTC-3'
Dre PR Rev	5'-GATAGTTCCAGCCCTCCCTCTCTCTC-3'
Dre PR Fw1 NheI	5'-CGGCTAGCCGCATACGGTCTAGTCGCAG-3'
Dre PR Fw2 NheI	5'-CGGCTAGCCCTAGCGTGGACTTTACG-3'
Dre PR Rev1 BglII	5'-CGAGATCTGATAGTTCCAGCCCTCCCTC-3'
Dre PR Rev3 BglII	5'-CGAGATCTAAGGTTTGATGGGACCCTTGG-3'
Hsa PR Fw	5'-GGAGGCAAGAGCACCCACAAACACTTC-3'
Hsa PR Rev	5'-CTATGTACTTAAAATCCTCTCCCG-3'
Hsa PR Fw1 NheI	5'-CGGCTAGCCCCACAAACACTTCAGTTAAC-3'
Hsa PR Fw2 NheI	5'-CGGCTAGCGGAACTAATTACTCATGTAGA-3'
Hsa PR Rev1 BglII	5'-GAAGATCTCTATGTACTTAAAATCCTCTC-3'
Hsa PR Rev2 BglII	5'-GAAGATCTGGGATGATGTCACCCTGG-3'
**Primers used for cloning of zebrafish transcription factors**	
Dre Ets1a Fw	5'-GACAGCGGATCTTGTTGAGG-3'
Dre Ets1a Rev	5'-CAGTGTTGGAATGTGACTGACGC-3'
Dre Ets1a BamHI Fw	5'- CGGGATCCCACCATGACGGCAGCTGTCGATA-3'
Dre Ets1a NehI Rev	5'-CGGCTAGCTTACTCGTCCGTGTCGGG-3'
Dre Twist1a Fw	5'- GGTGATGTTTGAGGAAGAGGCGATGC-3'
Dre Twist1a Rev	5'-CCGTGCGTTAGTGAGATGTTGACATGG-3'
Dre Twist1a BamHI Fw	5'-CGGGATCCCACCATGTTTGAGGAAGAGGCGAT-3'
Dre Twist1a NehI Rev	5'-CGGCTAGCTTAGTGAGATGTTGACAT-3'
**qPCR primers**	
Mmu Hprt1 Fw	5´-AGCCAAATACAAAGCCTAAGATGAGCG-3'
Mmu Hprt1 Rev	5'-TCTGGGGACGCAGCAACTGACATTTC-3'
Mmu Hprt6 Fw	5'-GGTGGATATGCCCTTGACTATAATGA-3'
Mmu Hprt6 Rev	5'-CAACATCAACAGGACTCCTCCTATT-3'
Mmu Gapdh Fw	5'-CCTTCCGTGTTCCTACCCCCAATGT-3'
Mmu Gapdh Rev	5'-AGTGTAGCCCAAGATGCCCTTCAGT-3'
Mmu Tnap Fw	5'-ACAACCTGACTGACCCTTCGCTCTCCG-3'
Mmu Tnap Rev	5'-CCAGCCAAAGATGTGGAGTTGCCCCG-3'
Mmu Oc Fw	5'-AAGCAGGAGGGCAATAAGGTAGTGAACA-3'
Mmu Oc Rev	5´-GAGTTTGGCTTTAGGGCAGCACAGGTC-3'
Mmu Mgp Fw	5'-ACACAGAGGCAGACTCACAGGACACCC-3'
Mmu Mgp Rev	5'-CTGAGGGGACATAAAGGTGTTGGCAT-3'
Mmu Sox9 Fw	5'-AGGTGCTGAAGGGCTACGACTGGACG-3'
Mmu Sox9 Rev	5'-GCTGGTACTTGTAATCGGGGTGGTCTT-3'
Mmu Col2a1 Fw	5'-AAGTGGGGCAAGACCGTCATCG-3'
Mmu Col2a1 Rev	5'-AGGGGAGGACGGTTGGGTATCA-3'
Mmu Col10a1 Fw	5'-TGGGATGCCGCTTGTCAGTGCTAAC-3'
Mmu Col10a1 Rev	5'-ATCCAGGTAGCCTTTGCTGTACTCATCATA-3'
Mmu Sp7 Fw	5'-TCCTATGCTCCGACCTCCTCAACTTTT-3'
Mmu Sp7 Rev	5'-GGAAGCAGAAAGATTAGATGGCAACGAG-3'
Mmu Atf4 Fw	5'-GTGTTGGCGGGGGACTTGATGT-3'
Mmu Atf4 Rev	5'-TCTCCAACATCCAATCTGTCCCG-3'
Mmu Ptch1 Fw	5'-TGGGGGTTCTCAATGGACTGGT-3'
Mmu Ptch1 Rev	5'-CGAGTCGGAGGAATCAGACCCATT-3'
Dre 18S Fw	5'-AACACGAACATTGATGGAAGACG-3'
Dre 18S Rev	5'-ATTAGCAAGGACCTGGCTGTATTT-3'
Dre ef1α Fw	5'-ACGCCCTCCTGGCTTTCACCC-3'
Dre ef1α Rev	5'-TGGGACGAAGGCAACACTGGC-3'
Dre atf4a Fw	5'-CAGAGTCGCCTACCCTTACA-3'
Dre atf4a Rev	5'-CCCTCTTGACTTCTTCCATTAGC-3'
Dre atf4b Fw	5'-CAAGATGAGCACACTGAGGTTC-3'
Dre atf4b Rev	5'-CGGGTCTGGAGTATGGTTTAG-3'
Dre col2a1a Fw	5'-CAGGAAGAGTTTGGCGGCTGT-3'
Dre col2a1a Rev	5'-GACACGGCACGGTTCTGGTT-3'
Dre col10a1a Fw	5'-TGGAGCGCCTGGAGTTGGTT-3'
Dre col10a1a Rev	5'-GGCCCAGATTCCCCATCACGG-3'
Dre mgp Fw	5'-CGCAGATACTGAGCCCCGCCT-3'
Dre mgp Rev	5'-CATGAAGCGTGTCCAGGGTTTATTGA-3'
Dre sox9a Fw	5'-CGTCCATCTACGGTGTTCGCAT-3'
Dre sox9a Rev	5'-CGGACGGGCAGGGCGA-3'
Dre sox9b Fw	5'-CATCCAGACTACAAATACCAGCC-3'
Dre sox9b Rev	5'-GCTGGAGTCGGGTGTTTCT-3'
Dre sox10 Fw	5'-TCACGCTACAGGTCAGAGTCA-3'
Dre sox10 Rev	5'-ATTTCGCCAATGTCCACG-3'
Dre Runx2a Fw	5'-AGCCGACCCACGCCAGTTTGAG-3'
Dre Runx2a Rev	5'-TGGGGTGTAGGTGAATGTTGCTGGATA-3'
Mmu U6 Fw	5'-AGGATGACACGCAAATTCGTG-3'
Mmu miR-214 Fw	5'-ACAGCAGGCACAGACAGGCAG-3'
Mmu miR-199a Fw	5'-CCCAGTGTTCAGACTACCTGTTC-3'
Dre U6 Fw	5'-AGGATGACACGCAAATCCGTG-3'
Dre miR-214 Fw	5'-ACAGCAGGCACAGACAGGCAG-3'
Dre miR-199a Fw	5'-CCCAGTGTTCAGACTACCTGTTC-3'
Dnm3os pomoter ChIP Fw	5'-CATTGCCAGTTCCCTAGTCTGCTGC-3'
Dnm3os promoter ChIP Rev	5'-CGACTGAGACACACTAAAGGGCTG-3'
**Comercial primers**	
oligo-d(T)-adapter primer	5'-ACGCGTCGACCTCGAGATCGATG(T)13-3'
universal adapter	5'-ACGCGTCGACCTCGAGATCGATG-3'
AP1 (Marathon library specic primer)	5´-CCATCCTAATACGACTCACTATAGGGC-3'
AP2 (Marathon library specic primer)	5´-ACTCACTATAGGGCTCGAGCGGCCGCCCGGGCAGGT-3'
**LNA probes**	
dre-miR-214 probe	DIG-5'-CTGCCTGTCTGTGCCTGCTGT-3'
Scrambled probe	DIG-5'-GTGTAACACGTCTATACGCCCA-3'

Supplementary Table S1. List of primers and oligoduplexes used in this study.
